# *Cistanche* Species Mitogenomes Suggest Diversity and Complexity in *Lamiales*-Order Mitogenomes

**DOI:** 10.3390/genes13101791

**Published:** 2022-10-04

**Authors:** Yujing Miao, Haimei Chen, Wanqi Xu, Chang Liu, Linfang Huang

**Affiliations:** Key Laboratory of Chinese Medicine Resources Conservation, State Administration of Traditional Chinese Medicine of the People’s Republic of China, Institute of Medicinal Plant Development, Chinese Academy of Medical Sciences & Peking Union Medical College, Beijing 100193, China

**Keywords:** *Cistanche*, mitogenome, MTPT, repeat, segment duplication, multi-copy PCGs, substitution rate

## Abstract

The extreme diversity and complexity of angiosperms is well known. Despite the fact that parasitic plants are angiosperms, little is known about parasitic plant mitogenomic diversity, complexity, and evolution. In this study, we obtained and characterized the mitogenomes of three *Cistanche* species (holoparasitic plants) from China to compare the repeats, segment duplication and multi-copy protein-coding genes (PCGs), to clarify the phylogenetic and evolution relationship within the *Lamiales* order, and to identify the mitochondrial plastid insertions (MTPT) in *Cistanche* mitogenomes. The results showed that the mitogenome sizes of the three *Cistanche* species ranged from 1,708,661 to 3,978,341 bp. The *Cistanche* species genome encodes 75–126 genes, including 37–65 PCGs, 31–58 tRNA genes and 3–5 rRNA genes. Compared with other *Lamiales* and parasitic species, the *Cistanche* species showed extremely high rates of multi-copy PCGs, ranging from 0.13 to 0.58 percent of the total number of PCGs. In addition, 37–133 Simple Sequence Repeat (SSRs) were found in these three mitogenomes, the majority of which were the mononucleotides Adenine/Thymine. The interspersed repeats contained forward and palindromic repeats. Furthermore, the segment-duplication sequence size ranged from 199,584 to 2,142,551 bp, accounting for 24.9%, 11.7% and 53.9% of the *Cistanche deserticola*, *Cistanche salsa* and *Cistanche tubulosa* mitogenome, respectively. Furthermore, the Ka/Ks analysis suggested that the *atp*4, *ccmB*, *ccmFc* and *matR* were probably positively selected during *Lamiales* evolution. The *Cistanche* plastome suggested the presence of MTPT. Moreover, 6–12 tRNA, 9–15 PCGs fragments and 3 rRNA gene fragments in the *Cistanche* mitogenomes were found in the MTPT regions. This work reports the *Cistanche* species mitogenome for the first time, which will be invaluable for study the mitogenome evolution of *Orobanchaceae* family.

## 1. Introduction

The *Cistanche* species are a group of non-photosynthetic parasitic plants. *Cistanche* is an Old World genus with about two dozen species. They have been divided into four well supported and geographically differentiated clades: East Asian Clade, Northwest African Clade, Southwest Asian Clade and Widespread Clade [1]. The *Cistanche* genus belongs to the *Orobancheaceae* family. The *Orobanchaceae* family contains about 2000 species (mostly parasitic plants) in 90–115 genera, and it belongs to *Lamiales* order [2]. Depending on the need of a host to complete its life cycle, *Orobancheaceae* plants are typically classified as obligate and facultative parasites. In addition, depending on their photosynthesis ability, *Orobancheaceae* plants are divided into a group with photosynthetic activity (hemiparasites) and a group with no photosynthetic activity (holoparasites) [3]. The *Orobanchaceae* family is an excellent model system for studying the evolution of parasitism in plants [4].

Previous studies showed that the genome size, genome structure, and gene content of *Orobanchaceae* (parasitic plants) plastomes vary dramatically compared with other angiosperm plants [5,6]. Furthermore, severe gene loss and pseudogenization within *Orobanchaceae* species plastomes were found [7]. To date, 46 complete *Orobanchaceae* plastid genomes have been deposited in GenBank, while only one *Orobanchaceae* mitogenome has been deposited. The complete mitogenome of an *Orobanchaceae* species, *Castilleja paramensis*, has the lowest gene-loss rate (7.69%) among the seven *Orobanchaceae* species. The low level of gene loss in the mitogenomes suggests that the parasitic plants still have a typical mitochondrial function [8].

The first complete mitogenome from parasitic mistletoes also exhibited significant degradation [9]. In addition, the parasitic mitogenomes about which previous reports were published were highly divergent in many aspects, including genome size, genome structure, gene loss, etc. For example, the *Viscum scurruloideum* mitogenome was 66 kb in size, with one circular and one linear chromosome [9]. The *Gastrodia elata* mitogenome was 20 times larger than that of *V. scurruloideum*, and it contained 12 circular and 7 linear chromosomes [10]. It is unknown, however, whether this observation applies generally to other parasitic plants in terms of genome structures.

Mitochondria are organelles that participate in a variety of metabolic activities linked to energy generation, synthesis and destruction [11]. The most well-known function of mitochondria is oxidative phosphorylation, which uses the proton gradient to produce ATP for metabolic activity [12]. In general, the mitogenome represents mitochondrial genetic information. Compared to those of animals and fungi, plant mitogenomes display many unique features. The sizes of the mitogenomes in angiosperms range from 200 kb to 11 Mb, and differ greatly among species [9,13]. Most angiosperm mitogenomes contain 24 to 41 PCGs and 2 or 3 rRNA genes [14,15,16,17]. The mitogenome expansion is largely fueled by DNA duplications and the insertion of foreign DNA, such as nuclear DNA, plastid-derived DNA (referred to as mitochondrial plastid insertions, MTPTs), and even horizontal gene transfers (HGTs) [15,18,19].

In addition, the synonymous substitution rates are typically quite low in plant mitogenomes and are similar among PCGs. A surprising amount of synonymous rate variation was also seen in some plants’ mitochondrial PCGs [20]. The variability of the above mitochondrial sequences and PCGs has sparked researchers’ interest in the mitogenome. To date, 8592 complete plastomes have been deposited in GenBank, while only 457 complete plant mitogenomes have been deposited. Therefore, it is necessary to study plant mitogenomes.

Most *Cistanche* species are traditionally used as medicinal herbs. In particular, *C. deserticola* is known as desert ginseng for its nourishing effects [21]. Several studies reported the chemical components and pharmacological effects of *Cistanche* species [22,23,24]. Previously, we reported the features of four *Cistanche* plastomes in China, and we found that the *Cistanche* plastomes were significantly different from those of other angiosperms [25]. By contrast, the study of the mitogenomes of *Cistanche* species is still in its infancy.

In this work, for the first time, we sequenced, assembled and characterized three full *Cistanche* species mitogenomes to: (1) remedy the lack of knowledge of *Cistanche* mitogenomes; (2) explore the complexity and diversity of *Cistanche* mitogenomes in terms of genome size, multi-copy PCGs, repeat, segment duplication, phylogenetic relationship and substitution rate; and (3) analyze the MTPT in the *Cistanche* mitogenomes. The results from this study will provide invaluable information for *Cistanche*-mitogenome evolution.

## 2. Results

### 2.1. Characteristics of the Cistanche Mitogenomes

The three *Cistanche* mitogenomes contained multiple linear chromosomes (Table 1; Figures S1–S3). The lengths of the *C. deserticola*, *C. salsa* and *C. tubulosa* mitogenomes were 1,860,774 bp, 1,708,661 bp and 3,978,341 bp, respectively (Table 1). Compared with *C. deserticola* and *C. salsa*, the *C. tubulosa* mitogenome was significantly larger. The overall GC contents of the *C. deserticola*, *C. salsa* and *C. tubulosa* mitogenomes were 44.59%, 44.52% and 44.57%, respectively (Table 1).

We annotated the three *Cistanche* species mitogenomes. The functional and structural classification of the annotated genes are shown in Table 2. According to our annotation, the *C. deserticola* mitogenome contained 82 genes, including 37 PCGs, 39 tRNA and 5 rRNA. The *C. salsa* mitogenome contained 75 genes, including 41 PCGs, 31 tRNA and 3 rRNA. The *C. tubulosa* mitogenome contained 126 genes, including 65 PCGs, 58 tRNA and 4 rRNA (Table 1). The functional genes were divided into ten classes, according to American College of Medical Genetics and Genomics (ACMG): Complex I (*nad*1, *nad*2, *nad*3, *nad*4, *nad*4L, *nad*5, *nad*6, *nad*7 and *nad*9); complex II (*sdh*3 and *sdh*4); complex III (*cob*); complex IV (*cox*1, *cox*2 and *cox*3); complex V (*atp*1, *atp*4, *atp*6, *atp*8 and *atp*9); cytochromec biogenesis (*ccmB*, *ccmC*, *ccmFc* and *ccmFn*); intron maturase (*matR*); secY independent transport (*mttB*); ribosomal protein large subunit (*rpl*2, *rpl*5, *rpl*10 and *rpl*16), and ribosomal protein small subunit (*rps*3, *rps*4, *rps*10, *rps*12, *rps*13 and *rps*14) (Table 2).

It is known that mitochondrial PCGs are variable in genetic structure. The functional genes were divided into three classes: non-intron-containing, trans-spliced and cis-spliced gene. Most PCGs belonging to the same functional categories shared similar genetic structures. We found that four, seven and eight PCGs were trans-spliced genes in *C. deserticola* (*ccmFc*, *cox*1, *nad*4 and *nad*7), *C. salsa* (*atp*1, *ccmFc*, *cob*, *nad*7, *rpl*2, *rps*3 and *rps*10) and *C. tubulosa* (*ccmFc*, *cox*1, *nad*4, *nad*5, *nad*7, *rpl*10, *rps*3 and *rps*12), respectively (Table 2). In addition, we found that three, four and two PCGs were cis-spliced genes in *C. deserticola* (*nad*1, *nad2* and *nad*5), *C. salsa* (*nad*1, *nad*2, *nad*4 and *nad*5) and *C. tubulosa* (*nad*1 and *nad*2), respectively (Table 2). Notably, the plastid PCGs (*rpl*16 and *rps*14) were likely to be two pseudogenes (Table 2).

### 2.2. Comparison of Multi-Copy Protein-Coding Genes (PCGs) in the Three Cistanche Species and Eight Other Lamiales and Six Parasitic Species Mitogenomes

**Figure 1:**
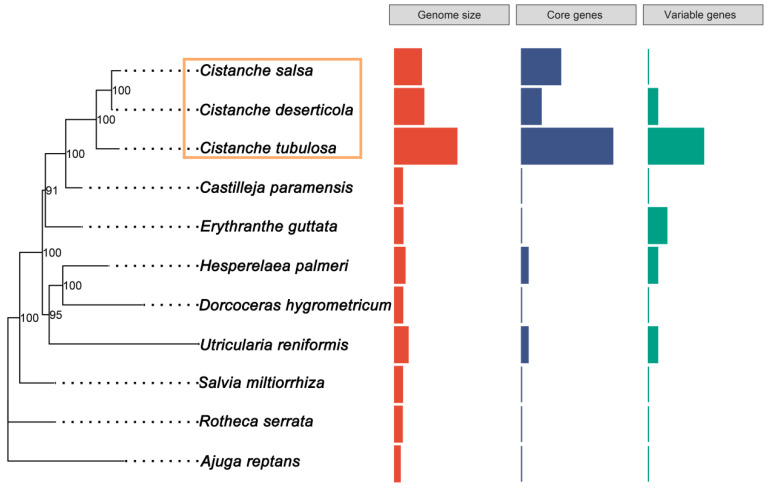
Genome size and protein-coding-gene contents among 11 *Lamiales* species mitogenomes.

We compared the mitogenome size, GC content and PCGs copy of the *Cistanche* species and other *Lamiales* species with published mitogeomes. The published *Lamiales* species included *Boea hygrometrica*, *Mimulus guttatus*, *Ajuga reptans*, *Salvia miltiorrhiza*, *Hesperelaea palmeri*, *Castilleja paramensis*, *Utricularia reniformis* and *Rotheca serrate* (Table S2). Their GC contents ranged from 43.27% to 45.5%, which were fairly similar (Table 1 and Table S2). However, the sizes of these mitogenomes were extremely variable (Figure 1, Table 1 and Table S2). The *C. tubulosa* mitogenome (3,978,341 bp) was the largest; its size was 11.3 times larger than the smallest mitogenome (*A. reptans*, 352,069 bp) (Table 1 and Table S2). The *Cistanche* mitogenomes were the largest among all the mitogenomes found in the *Lamiales* order. To determine whether there were any correlations between the PCG copy numbers and the mitogenome size, the PCGs copy numbers from those 11 mitogenomes were compared. The duplication of PCGs was observed in all the *Lamiales* mitogenomes. The degree of duplication was especially high in the *Cistanche* genus (Figure 1). The mitochondrial PCGs were divided into two categories, core genes and variable genes, according to a previous study [9]. Among the *Cistanche* mitogenomes, the proportion of duplicated core genes ranged from 13% to 58%, in the following order: *C. tubulosa* (58%), *C. salsa* (25%) and *C. deserticola* (13%) (Figure 1 and Table S4). The proportion of duplicated variable genes ranged from 0–35%, in the following order: *C. tubulosa* (35%), *C. deserticola* (6%) and *C. salsa* (0%) (Figure 1 and Table S4). Among other *Lamiales* species, the duplication of core genes was only present in the *H. palmeri* and *U. reniformis* mitogenomes (Figure 1). Furthermore, the duplication of variable genes was present in the *E. guttata*, *H. palmeri* and *U. reniformis* mitogenomes (Figure 1).

We also compared the size, GC content and number of PCG copies of the mitogenomes from the *Cistanche* species and several parasitic plants, including *C. paramensis*, *Cynomorium coccineum*, *Epirxanthes elongate*, *Lophophytum mirabile*, *Viscum album* and *V. scurruloideum* (Table S5). Their GC contents ranged from 43.52% to 47.4%, which were fairly similar (Table 1 and Table S5). However, the sizes of these mitogenomes varied greatly (Figure S4, Table 1 and Table S5). The smallest mitogenome was from *V. scurruloideum* (65,873 bp). The *Cistanche* mitogenomes remained the largest among the parasitic plants (Figure S4, Table 1 and Table S5). It is worth noting that the mitogenome size of *C. coccineum*, a holoparasitic plant, was also over 1 Mb (Table S5).

In addition to *C. paramesis*, *V. scurruloideum* and *V. album*, the duplication of PCGs was also present in the mitogenomes of the parasitic plants (Figure S4 and Table S6). Among the *Cistanche* mitogenomes, the proportion of duplicated core genes ranged from 13% to 0.63%, in the following order: *C. tubulosa* (63%), *C. salsa* (25%) and *C. deserticola* (13%) (Figure S4 and Table S6). Furthermore, the proportion of duplicated variable genes ranged from 0–35%, in the following order: *C. tubulosa* (35%), *C. deserticola* (6%) and *C. deserticola* (0) (Figure S4 and Table S6). Overall, *C. tubulosa* and *C. coccineum* had the highest proportions of duplicated core genes and variable genes (Figure S4 and Table S6).

### 2.3. Identification of MTPTs

To identify the MTPTs in the *Cistanche* mitogenomes, we compared the *Cistanche* mitogenome sequences with their plastome sequences. For *C. deserticola*, 158 high-scoring segment pairs (HSPs) were found. These 158 fragments were 35,165 bp in length in total, accounting for 1.89% of the mitogenome length (Figure S5). The fragment length ranged from 33 to 1156 bp. The annotated results showed that they were all plastid genes, including 11 complete tRNA genes: *trn*V-GAC (2), *trn*N-GUU, *trn*M-CAU, *trn*W-CCA (2), *trn*S-GGA (2), *trn*S-GCU (2) and *trn*S-GGA. Furthermore, nine fragments were annotated as partial plastid PCGs: *rpl*2 (170 bp), *ycf*2 (276, 387, 216, 61, 36, 61 bp), *rps*14 (199 bp) and *rps*4 (193 bp). The remaining fragments were identified as plastid ribosome RNAs (*rrn* 5, 16S and 23) (Table S7).

For *C. salsa*, 128 HSPs were identified. These 128 fragments were 28,963 bp in length in total, accounting for 1.64% of the mitogenome length (Figure S6). The fragment length ranged from 33 to 1155 bp. The annotated results showed that they were all plastid genes, including 12 complete tRNA genes: *trn*S-GGA (5), *trn*N-GUU (2), *trn*D-GUC (2), *trn*W-CCA, *trn*M-CAU and *trn*V-GAC. Furthermore, nine fragments were annotated as partial plastid PCGs: *rps*4 (193 bp), *rps*14 (199,193 bp) and *ycf*2 (216, 170, 61, 387, 276, 396 bp). The remaining fragments were identified as plastid ribosome RNAs (*rrn* 5, 16S and 23) (Table S8).

For *C. tubulosa*, 139 HSPs were found. These 139 fragments were 26, 911 bp in length in total, accounting for 0.68% of the mitogenome length (Figure S7). The fragment length ranged from 35 to 1156 bp. The annotated results showed that they were all plastid genes, including six complete tRNA genes: *trn*D-GUC (2), *trn*N-GUU, *trn*D-GUC, *trn*N-GUU and *trn*R-ACG. Furthermore, 15 fragments were annotated as partial plastid PCGs: *ycf*2 (128, 81, 110, 81, 257, 110, 110, 128, 77 bp), *ycf*1 (580, 580 bp), *accD* (243, 31, 243 bp) and *rpl*2 (138 bp). The remaining fragments were identified as plastid ribosome RNA (*rrn* 5, 16S and 23) (Table S9).

### 2.4. Repeats and Segment Duplication Analysis

**Figure 2:**
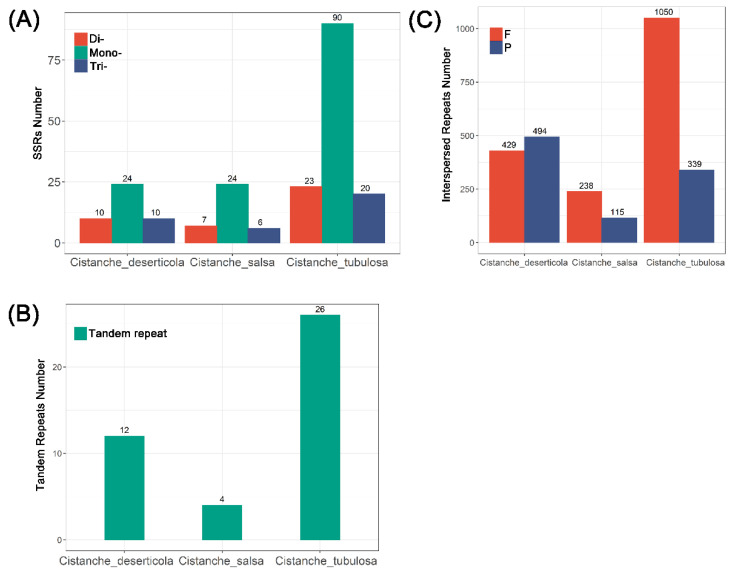
Simple sequence repeats (SSRs), interspersed repeats and tandem repeats in 3 *Cistanche* species mitogenomes. (**A**) Comparison of SSRs among the three mitogenomes. Each color column represents a different SSR-repeat type. (**B**) Comparison of tandem repeats among the three mitogenomes. (**C**) Comparison of interspersed repeats in the three mitogenomes. Each color column represents a different interspersed-repeat type. The number of repeats in each category is shown on the top of the corresponding columns.

The types and numbers of repeats varied among the three mitogenomes. SSRs are sequences composed of repeats with motifs 1 to 6 bp in length. Among the *Cistanche* mitogenomes, the number of SSRs ranged from 37–133 in the following order: *C. tubulosa* (133), *C. deserticola* (44) and *C. salsa* (37). Polyadenine or polythymine repeat types were the most prevalent mononucleotide SSRs (Figure 2A and Table S10). This result was in agreement with the fact that the AT content (55.41–55.43%) was higher than the GC content (44.57–44.59%) in the *Cistanche* mitogenomes (Table 1).

Next, we detected the interspersed repeats by REPuter. The interspersed repeats were divided into four types: forward, palindrome, reverse and complement repeats. In the *Cistanche* mitogenomes, the forward and palindromic repeats were the main types of interspersed repeats (Figure 2B and Tables S11–S13). Only 353 interspersed repeats were detected in the *C. salsa* mitogenome (Table S12), and more than 1300 in the *C. tubulosa* (Table S13).

In addition to the SSRs and interspersed repeats, we also detected tandem repeats >30 bp in length and similarities of >90%. The number of interspersed repeats ranged from 4 to 26 in the *Cistanche* mitogenomes (Figure 2C). The number of repeat units ranged from 1.8 to 2.5 copies per tandem repeat, and the repeat sizes ranged from 21 to 127 bp (Tables S14–S16).

**Figure 3:**
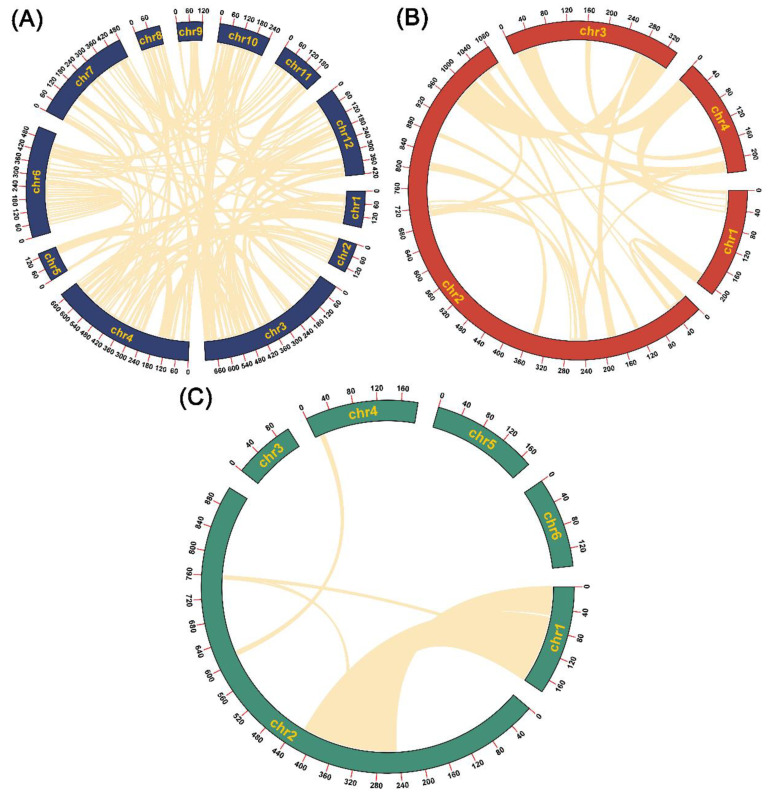
The mitogenomic-segment-duplication distributions of the 3 *Cistanche* species. (**A**) *Cistanche tubulosa*. (**B**) *Cistanche deserticola*. (**C**) *Cistanche salsa*. The outermost circle marks the position of the mitogenome. The bright yellow arcs indicate segment duplication.

The segment-duplication-identification results showed that the segment sequences ranged from 199,584 bp to 2,142,551 bp in length (Table 1), accounting for 24.9%, 11.7% and 53.9% of the lengths of the *C. deserticola*, *C. salsa* and *C. tubulosa* mitogenomes, respectively (Figure 3). In the *C. deserticola* mitogenome, 39 alignments were identified. The lengths of the alignments ranged from 5078 bp to 38,025 bp (Table S17). By contrast, only 14 alignments were identified in the *C. salsa* mitogenome. The lengths of the alignments ranged from 5385 bp to 23,085 bp (Table S18). It is worth noting that 168 alignments were found in the *C. tubulosa* mitogenome. The lengths of the alignments ranged from 5169 bp to 64,106 bp (Table S19). These repeat sand segment duplications might have promoted genome rearrangement and contributed to the variations in genome size.

### 2.5. Phylogenetic Analysis by Mitogenome Sequences

**Figure 4:**
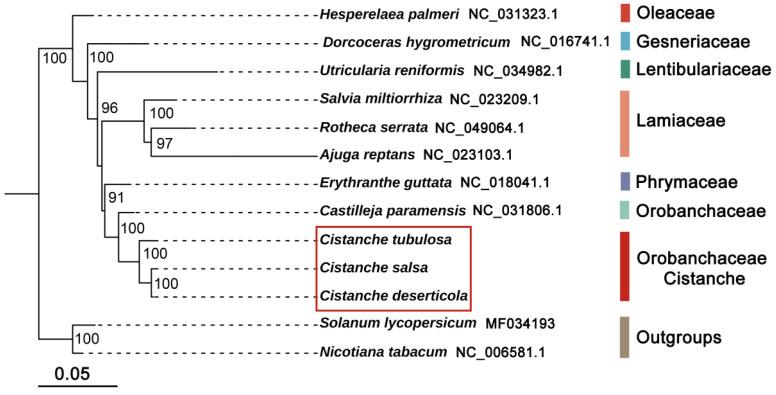
Phylogenetic relationships of *Cistanche* species with 8 other *Lamiales* species. The tree was constructed based on the amino-acid sequences of 49 mitochondrial protein-coding genes, including *atp*1, *atp*4, *atp*6, *atp*8, *atp*9, *atp*B, *atp*E, *ccmB*, *ccmC*, *ccmFc*, *ccmFn*, *cob*, *cox*1, *cox*2, *cox*3, *cyt*B, *Itr*A, *matR*, *mttB*, *nad*1, *nad*2, *nad*3, *nad*4, *nad*4L, *nad*5, *nad*6, *nad*7, *nad*9, *pet*G, *pet*L, *rbc*L, *rpl*2, *rpl*5, *rpl*10, *rpl*16, *rpl*23, *rpl*36, *rps*1, *rps*3, *rps*4, *rps*7, *rps*10, *rps*11, *rps*12, *rps*13, *rps*14, *rps*19, *sdh*3 and *sdh*4. Species in red box were three *Cistanche* species.

The phylogeny was reconstructed using shared mitochondrial PCGs from 11 *Lamiales* mitogenomes using the maximum-likelihood (ML) method. The sister genus of *Cistanche* was *Castilleja*, with a bootstrap score (BS) of 100 (Figure 4). These two genera belong to the *Orobanchaceae* family. The species of *Cistanche* were distributed in two main clades. The first clade (BS: 100) was formed by *C. deserticola* and *C. salsa*, with the same mitogenome size. The second clade contained *C. tubulosa* with a BS of 100. These two clades were subsequently clustered together (BS: 100) (Figure 4). The bootstrap scores were high for all the branches, indicating the high degree of reliability of the phylogenetic tree. Moreover, the phylogenetic relationship of the *Cistanche* species constructed using the mitogenomes was congruent with that using the plastid genome, as shown in our previous studies.

### 2.6. The Substitution Rate of Mitochondrial PCGs

The shared mitochondrial PCGs were used to estimate the nucleotide substitution rate of the mitochondrial PCGs in *Lamiales*. For each of the 28 PCGs, the pairwise Ka/Ks ratios were calculated. We found that the Ka/Ks rations of the four PCGs were over 1.0 in most of the species (Figure 5 and Table S20). These four PCGs were *atp*4, *ccmB*, *ccmFc* and *matR*, suggesting potential positive selection. However, most of the mitochondrial PCGs showed low Ka/Ks ratios, indicating possible purifying selection. In particular, the Ka/Ks ration of a*tp*9, *cox*1, *cox*3 and *nad*4L showed a relatively low value (Figures S8–S31).

## 3. Discussion

### 3.1. Genome Expansion in C. tubulosa

A substantial portion of plant mitogenomes may be made up of small repetitive sequences [26]. For example, low-complexity repetitive DNA made up 5–10% of the sequences in the *Citrullus* and *Cucurbita* mitogenomes [27]. Furthermore, similar ratios of low-complexity repetitive DNA sequences were observed in other plants [9,28]. In our study, *C. tubulosa*, *C. salsa* and *C. deserticola* had repeat sequences that were 174,618 bp, 32,375 bp and 149,945 bp long, accounting for 4.4%, 1.89% and 8.06% of the mitogenome sizes, respectively. These results were consistent with the results of previous studies, which showed that repeat sequences can cause changes in mitogenome size [9,26,27,28].

*C. deserticola* possessed the largest proportion of repetition sequences among the three *Cistanche* mitogenomes in our study. However, the mitogenome size of *C. derserticola* was close to that of *C. salsa*, which means that other factors may also play important roles in mitogenome size in addition to small repetitive sequence. Segment duplication and multi-copy protein-coding genes were two other factors causing mitogenome expansion.

In the *C. tubulosa* mitogenome, the sequences of the duplicated segments were 2,142,511 bp in length, accounting for 53.9% of the whole mitogenome size. Similarly, Sloan reported that 4.6-megabyte repeats were identified in the mitogenome of *Silene conica*, accounting for 40.8% of the whole mitogenome [13]. Furthermore, multi-copy protein-coding genes might result in mitogenome expansion. In the *C. tubulosa* mitogenome, the proportions of duplicated core genes and variable genes were 58% and 35%, respectively. For typical large mitogenomes (*Cucumis* species), some protein-coding genes (*rps19*) are also presented twice in both *Citrullus* and *Cucurbita* [14]. In *C. tubulosa*, complex Ⅰ (*nad*4, *nad*4L, *nad*6 and *nad*7), complex Ⅳ (*cox*1 and *cox*2), complex Ⅴ (*atp*4, *atp*8 and *atp*9), Cytochromec biogenesis (*ccmFc* and *ccmFn*), Ribosomal protein small subunit (*rps*3, *rps*4 and *rps*14), Intron maturase (*matR*) and SecY independent transport (*mttB*) protein-coding gene all had multiple copies. We speculated that this was related to the fact that holoparasitic plants do not conduct photosynthesis. In addition, environmental stress might up-regulate the expression of some related genes. Previous studies suggested that the plant mitochondrial electron-transport chain could improve plant performance under stressful environmental conditions [29]. Unlike *C. deserticola* and *C. salsa*, *C. tubulosa* experience salt stress and cold stress rather than drought stress [30,31,32]. This might lead to the duplication of genes in *C. tubulosa*. In summary, small repeats, segment duplication, and multi-copy genes were the main causes of the mitogenome expansion.

### 3.2. The Presence of MTPTs

In angiosperm plants, MTPTs are almost always present [33]. In our study, we found 158 *C. deserticola*, 128 *C. salsa* and 139 *C. tubulosa* MTPT sequences. They were 35,165 bp, 28,963 bp and 26,911 bp in length, accounting for 1.89%, 1.64% and 0.68% of the *C. deserticola*, *C. salsa* and *C. tubulosa* mitogenomes, respectively. Cheng et al. suggested that 26.87-kilobyte MTPT fragments were found in *Suaeda glauca*, accounting for 5.18% of the mitogenome [34]. In addition, the MTPTs discovered in *Salix suchowensis* account for 11.3% (17.5 kb) of the plastome and 2.8% (18.1 kb) of the mitogenome [35]. Interestingly, the proportion of MTPTs in the *Cistanche* mitogenomes was relatively low. In addition, our results showed that, in the MTPT sequences, the plastid PCGs and ribosome RNA were partial sequences. By contrast, the plastid tRNA genes had complete sequences in the MTPT fragments. The partial loss of plastid PCGs and ribosomal RNAs suggest that they might no longer function in the MTPT sequences. This supports the theory that fragments of DNA from plastomes usually become nonfunctional pseudogenes, while some tRNA genes still perform normal functions [36].

## 4. Materials and Methods

### 4.1. Sampling, DNA Extraction, and Genome Sequencing

Fresh samples of *C. deserticola*, *C. salsa* and *C. tubulosa* were collected from the Alxa League (Inner Mongolia Autonomous Region), Tacheng City (Xinjiang Uygur Autonomous Region), Hotan Prefecture (Xinjiang Uygur Autonomous Region), China (Table S1). The samples were identified by Professor Yulin Lin and stored at the Herbarium of the Chinese Academy of Medical Science and Peking Union Medicinal College (under specimens registry numbers CMPB13484, CMPB13485 and CMPB13487). Total DNA extraction was carried out using a plant genomic DNA extraction kit (Tiangen Biotech, Beijing, China). Utilizing NEBNext^®^ library building kit [37], a DNA library with an insert size of 400 bp was constructed. Subsequently, Illumina HiSeq4000 sequencing platform was used for sequencing. The sequencing produced 5.44, 5.16 and 4.34 G of raw data, respectively (Table S1). Trimmomatic was used to filter the raw data to obtain the clean data [38]. In total, 4.88 G, 4.62 G and 3.92 G clean data were obtained, respectively. The plant samples were also used for Oxford Nanopore sequencing. Library construction, quality detection and sequencing were conducted following the manufacturer’s standard protocol. Consequently, 78.96 G, 39.19 G and 59.52 G of raw data were obtained, and 48 G, 31.96 G and 54.56 G remained after filtering and qualification (Table S1).

### 4.2. Mitogenome Assembly and Annotation

Eight *Lamiales* mitogenomes were downloaded as references from NCBI (Table S2). We initially enriched the mitogenome-related clean reads from Oxford Nanopore data using USEARCH [39]. The filtered Nanopore reads were assembled into contigs using Nextdenovo v2.4.0 (available online: https://github.com/Nextomics/NextDenovo (20 December 2020)) with the default parameters. The obtained contigs were then used as references, named *Cistanche* Structure Contigs. Illumina paired-end reads were mapped back to the *Cistanche* Structure Contigs using Minimap2 [40] and SAMtools [41]. We extracted the filtered Illumina paired-end reads and assembled them into contigs using SPAdes v. 3.10.1 [42]. By comparing the assembly of short-reads and long-reads using Minimap2 [40], we preliminarily determined which contig was the putative mitochondrial molecule. The assembly contigs obtained above were corrected with the Illumina paired-end reads using NextPolish1.3.1 [43]. Draft mitochondrial contigs were processed further following the steps below. Firstly, we compared the sequences with those in GenBank using BLASTn program to determine whether they were mitochondrial reads [44]. Second, we annotated the mitogenomes using MITOFY to determine whether the sequences contained mitochondrial genes [14].

### 4.3. Identification of Mitochondrial Plastid DNAs (MTPTs)

*Cistanche* mitogenomes were compared with *C. deserticola* (MN614127), *C. slsa* (MN614128) and *C. tubulosa* (MN614129) plastomes to identify MTPTs using BLASTn [44]. The BLASTn parameters was selected based on those reported previously [45]. Furthermore, TBtools was used to visualize the BLASTn results [46]. With the aid of *Cistanche* plastomes as a reference, the identified transferred DNA segments were annotated using BLASTn [44].

### 4.4. Analysis of Simple Sequence Repeats (SSRs), Tandem Repeats, Interspersed Repeats, and Segment Duplication

Online website MISA (Available online: http://webblast.ipk-gatersleben.de/misa/ (15 January 2021)) was used to identify the SSRs in mitochondrial genome. These SSRs included mono-, di-, tri-, tetra-, penta-, and hexanucleotides with minimum numbers of 10, 6, 5, 5, 5 and 5, respectively. With the default parameters, Tandem Repeat Finder [47] was used to identify tandem repeats. In addition, REPuter was used to identify forward, reverse, palindromic and complementary repeat sequences [48]. The minimum repeat size was set to 30 bp and the identity of the repeat units was ≥90%. Segment duplications were identified by comparing the mitochondrial genome to itself using BLASTN with the parameter setting e-value = 1 × 10^−5^. All alignments with length > 5000 bp and score > 90% were considered segment duplication for calculations of segment-duplication number. TBtools was used to visualize the BLASTn results [46].

### 4.5. Phylogenetic Analyses and Estimation of Nucleotide-Substitution Rates

For phylogenetic analyses, the DNA sequences of shared mitochondrial genome PCGs from 11 *Lamiales* species, including the *Cistanche* species in this work (Table S2), were used in the construction. The mitogenomes of other 8 *Lamiales* species were downloaded from GenBank Organelle Genome Resource database. PhyloSuite (v1.2.1) was used to extract shared mitochondrial PCGs from *Lamiales* species [49]. MAFFT (v7.450) was used to align the corresponding amino-acid sequences [50]. The aligned amino-acid sequences were concatenated and used to construct the phylogenetic trees through the maximum-likelihood (ML) method, using *Solanum lycopersicum* (MF034193) and *Nicotiana tabacum* (NC_006581.1) as outgroups. The bootstrap analysis was performed with 1000 replicates. We used the yn00 program in PAML v 4.9 [51] to calculate the nonsynonymous substitution rate (*d_N_*) and synonymous substitution rate (*d_S_*) for PCGs with the F3 × 4 codon model.

## 5. Conclusions

In conclusion, this study provides a first insight into the structural diversity and complexity of *Cistanche* mitogenomes. Our results answered the three scientific questions that were posed in the introduction. First, the complete mitogenomes of *C. deserticola*, *C. salsa* and *C. tubulosa* were successfully assembled, which was a significant accomplishment in the study of *Cistanche* mitogenomes. Second, the *C. tubulosa* was close to 4 Mb in size, indicating a significant expansion. Furthermore, the *C. tubulosa* mitogenomes differed significantly from those of the *C. deserticola* and *C. salsa* in terms of numbers of duplicated PCGs and segment duplication. Three *Cistanche* species were formed into one clade, close to the species of *Orobanchaceae*. Additionally, the topology of the *Lamiales* in the present study was highly similar to that in the APG IV system. Third, MTPT sequences were identified in three *Cistanche* species mitogenomes, with partial PCGs and ribosome RNA fragments, and complete tRNA from the plastomes. The results of this study therefore revealed many fascinating aspects of mitogenome diversity and complexity.

## Figures and Tables

**Figure 5:**
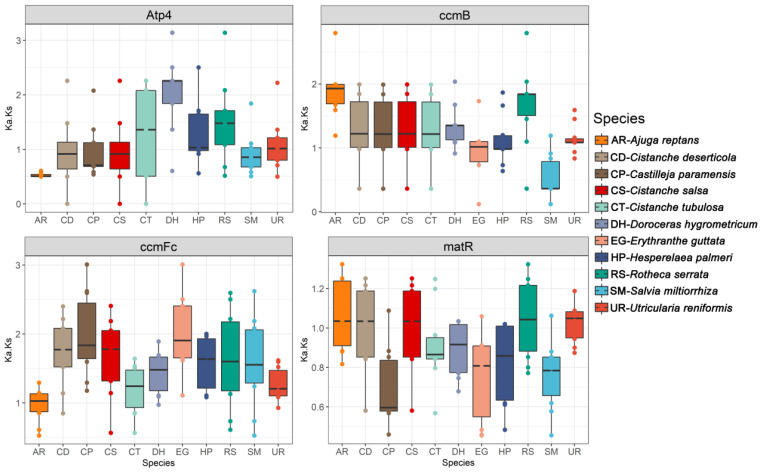
Boxplots of pairwise Ka/Ks values among each retained mitochondrial protein-coding gene within the 11 *Lamiales* species.

**Table 1:** Basic information on *Cistanche* species mitogenomes. In the row, Chromosomes, the number means the total number of chromosomes, while L denotes linear. Se-dup means segment duplication.

	*C. tubulosa*	*C. salsa*	*C. deserticola*
Size (bp)	3,978,341	1,708,661	1,860,774
Chromosomes	12 L	6 L	4 L
GC%	44.57	44.52	44.59
Genes	126	75	82
Protein coding	65	41	37
tRNA	58	31	39
rRNA	4	3	5
Se-dup-Number	168	14	39
Se-dup-Size (bp)	2,142,551	199,584	463,566

**Table 2:** Protein-coding genes in *Cistanche* species mitogenomes. ●: no intron containing gene present; Ө: trans-spliced gene present; ○: cis-spliced gene present; Ψ: pseudogene present; ×: gene absent.

Functional Classification	Gene	*C. tubulosa*	*C. salsa*	*C. deserticola*
Complex V	*atp*1	●	Ө	●
	*atp*4	●	●	●
	*atp*6	●	●	●
	*atp*8	●	●	●
	*atp*9	●	●	●
Cytochromec biogenesis	*ccmB*	●	●	●
	*ccmC*	●	●	●
	*ccmFc*	Ө	Ө	Ө
	*ccmFn*	●	●	●
Complex III	*cob*	●	Ө	●
Complex IV	*cox*1	Ө	●	Ө
	*cox*2	●	●	●
	*cox*3	●	●	●
Intron maturase	*matR*	●	●	●
SecY independent transport	*mttB*	●	●	●
Complex I	*nad*1	○	○	○
	*nad*2	○	○	○
	*nad*3	●	●	●
	*nad*4	Ө	○	Ө
	*nad*4L	●	●	●
	*nad*5	Ө	○	○
	*nad*6	●	●	●
	*nad*7	Ө	Ө	Ө
	*nad*9	×	●	●
Ribusomal protein large subunit	*rpl*2	×	Ө	×
	*rpl*5	●	●	●
	*rpl*10	Ө	●	●
	*rpl*16	Ψ	Ψ	×
Ribosomal protein small subunit	*rps*3	Ө	Ө	×
	*rps*4	●	●	×
	*rps*10	×	Ө	×
	*rps*12	Ө	●	●
	*rps*13	●	●	●
	*rps*14	Ψ	Ψ	Ψ
Complex II	*sdh*3	×	×	×
	*sdh*4	●	●	●

## Data Availability

The assembled chloroplast genomes of *C. deserticola*, *C. salsa* and *C. tubulosa* were deposited in GenBank with the accession numbers ON890398–ON890419.

## Supplementary Materials

The following supporting information can be downloaded at: https://www.mdpi.com/article/10.3390/genes13101791/s1. Figure S1: Mitochondrial genome map of *Cistanche deserticola*. The outside circle shows the GC content. The inside circle represents the protein coding gene, tRNA and rRNA; Figure S2: Mitochondrial genome map of *Cistanche salsa*. The outside circle shows the GC content. The inside circle represents the protein coding gene, tRNA and rRNA; Figure S3: Mitochondrial genome map of *Cistanche tubulosa*. The outside circle shows the GC content. The inside circle represents the protein coding gene, tRNA and rRNA; Figure S4: Genome size and protein-coding gene content of 9 parasitic plants mitochondrial genomes; Figure S5: The collinearity analysis among *C. deserticola* mitochondrial genome and plastid genome; Figure S6: The collinearity analysis among *C. salsa* mitochondrial genome and plastid genome; Figure S7: The collinearity analysis among *C. tubulosa* mitochondrial genome and plastid genome; Figure S8: Boxplots of pairwise Ka/Ks values in *Atp*1 gene within 11 *Lamiales* species. AR—*Ajuga reptans*; CD—*Cistanche deserticola*; CP—*Castilleja paramensis*; CS—*Cistanche salsa*; CT—*Cistanche tubulosa*; DH—*Doroceras hygrometricum*; EG—*Erythranthe guttata*; HP—*Hesperelaea palmeri*; RS—*Rotheca serrate*; SM—*Salvai miltiorrhiza*; UR—*Utricularia reniformis*; Figure S9: Boxplots of pairwise Ka/Ks values in *Atp*6 gene within 11 *Lamiales* species. AR—*Ajuga reptans*; CD—*Cistanche deserticola*; CP—*Castilleja paramensis*; CS—*Cistanche salsa*; CT—*Cistanche tubulosa*; DH—*Doroceras hygrometricum*; EG—*Erythranthe guttata*; HP—*Hesperelaea palmeri*; RS—*Rotheca serrate*; SM—*Salvai miltiorrhiza*; UR—*Utricularia reniformis*; Figure S10: Boxplots of pairwise Ka/Ks values in *Atp*8 gene within 11 *Lamiales* species. AR—*Ajuga reptans*; CD—*Cistanche deserticola*; CP—*Castilleja paramensis*; CS—*Cistanche salsa*; CT—*Cistanche tubulosa*; DH—*Doroceras hygrometricum*; EG—*Erythranthe guttata*; HP—*Hesperelaea palmeri*; RS—*Rotheca serrate*; SM—*Salvai miltiorrhiza*; UR—*Utricularia reniformis*; Figure S11: Boxplots of pairwise Ka/Ks values in *Atp*9 gene within 11 *Lamiales* species. AR—*Ajuga reptans*; CD—*Cistanche deserticola*; CP—*Castilleja paramensis*; CS—*Cistanche salsa*; CT—*Cistanche tubulosa*; DH—*Doroceras hygrometricum*; EG—*Erythranthe guttata*; HP—*Hesperelaea palmeri*; RS—*Rotheca serrate*; SM—*Salvai miltiorrhiza*; UR—*Utricularia reniformis*; Figure S12: Boxplots of pairwise Ka/Ks values in *ccmC* gene within 11 *Lamiales* species. AR—*Ajuga reptans*; CD—*Cistanche deserticola*; CP—*Castilleja paramensis*; CS—*Cistanche salsa*; CT—*Cistanche tubulosa*; DH—*Doroceras hygrometricum*; EG—*Erythranthe guttata*; HP—*Hesperelaea palmeri*; RS—*Rotheca serrate*; SM—*Salvai miltiorrhiza*; UR—*Utricularia reniformis*; Figure S13: Boxplots of pairwise Ka/Ks values in *ccmFn* gene within 11 *Lamiales* species. AR—*Ajuga reptans*; CD—*Cistanche deserticola*; CP—*Castilleja paramensis*; CS—*Cistanche salsa*; CT—*Cistanche tubulosa*; DH—*Doroceras hygrometricum*; EG—*Erythranthe guttata*; HP—*Hesperelaea palmeri*; RS—*Rotheca serrate*; SM—*Salvai miltiorrhiza*; UR—*Utricularia reniformis*; Figure S14: Boxplots of pairwise Ka/Ks values in *cob* gene within 11 *Lamiales* species. AR—*Ajuga reptans*; CD—*Cistanche deserticola*; CP—*Castilleja paramensis*; CS—*Cistanche salsa*; CT—*Cistanche tubulosa*; DH—*Doroceras hygrometricum*; EG—*Erythranthe guttata*; HP—*Hesperelaea palmeri*; RS—*Rotheca serrate*; SM—*Salvai miltiorrhiza*; UR—*Utricularia reniformis*; Figure S15: Boxplots of pairwise Ka/Ks values in *cox*1 gene within 11 *Lamiales* species. AR—*Ajuga reptans*; CD—*Cistanche deserticola*; CP—*Castilleja paramensis*; CS—*Cistanche salsa*; CT—*Cistanche tubulosa*; DH—*Doroceras hygrometricum*; EG—*Erythranthe guttata*; HP—*Hesperelaea palmeri*; RS—*Rotheca serrate*; SM—*Salvai miltiorrhiza*; UR—*Utricularia reniformis*; Figure S16: Boxplots of pairwise Ka/Ks values in *cox*2 gene within 11 *Lamiales* species. AR—*Ajuga reptans*; CD—*Cistanche deserticola*; CP—*Castilleja paramensis*; CS—*Cistanche salsa*; CT—*Cistanche tubulosa*; DH—*Doroceras hygrometricum*; EG—*Erythranthe guttata*; HP—*Hesperelaea palmeri*; RS—*Rotheca serrate*; SM—*Salvai miltiorrhiza*; UR—*Utricularia reniformis*; Figure S17: Boxplots of pairwise Ka/Ks values in *cox*3 gene within 11 *Lamiales* species. AR—*Ajuga reptans*; CD—*Cistanche deserticola*; CP—*Castilleja paramensis*; CS—*Cistanche salsa*; CT—*Cistanche tubulosa*; DH—*Doroceras hygrometricum*; EG—*Erythranthe guttata*; HP—*Hesperelaea palmeri*; RS—*Rotheca serrate*; SM—*Salvai miltiorrhiza*; UR—*Utricularia reniformis*; Figure S18: Boxplots of pairwise Ka/Ks values in *mtt*B gene within 11 *Lamiales* species. AR—*Ajuga reptans*; CD—*Cistanche deserticola*; CP—*Castilleja paramensis*; CS—*Cistanche salsa*; CT—*Cistanche tubulosa*; DH—*Doroceras hygrometricum*; EG—*Erythranthe guttata*; HP—*Hesperelaea palmeri*; RS—*Rotheca serrate*; SM—*Salvai miltiorrhiza*; UR—*Utricularia reniformis*; Figure S19: Boxplots of pairwise Ka/Ks values in *nad*1 gene within 11 *Lamiales* species. AR—*Ajuga reptans*; CD—*Cistanche deserticola*; CP—*Castilleja paramensis*; CS—*Cistanche salsa*; CT—*Cistanche tubulosa*; DH—*Doroceras hygrometricum*; EG—*Erythranthe guttata*; HP—*Hesperelaea palmeri*; RS—*Rotheca serrate*; SM—*Salvai miltiorrhiza*; UR—*Utricularia reniformis*; Figure S20: Boxplots of pairwise Ka/Ks values in *nad*2 gene within 11 *Lamiales* species. AR—*Ajuga reptans*; CD—*Cistanche deserticola*; CP—*Castilleja paramensis*; CS—*Cistanche salsa*; CT—*Cistanche tubulosa*; DH—*Doroceras hygrometricum*; EG—*Erythranthe guttata*; HP—*Hesperelaea palmeri*; RS—*Rotheca serrate*; SM—*Salvai miltiorrhiza*; UR—*Utricularia reniformis*; Figure S21: Boxplots of pairwise Ka/Ks values in *nad*3 gene within 11 *Lamiales* species. AR—*Ajuga reptans*; CD—*Cistanche deserticola*; CP—*Castilleja paramensis*; CS—*Cistanche salsa*; CT—*Cistanche tubulosa*; DH—*Doroceras hygrometricum*; EG—*Erythranthe guttata*; HP—*Hesperelaea palmeri*; RS—*Rotheca serrate*; SM—*Salvai miltiorrhiza*; UR—*Utricularia reniformis*; Figure S22: Boxplots of pairwise Ka/Ks values in *nad*4 gene within 11 *Lamiales* species. AR—*Ajuga reptans*; CD—*Cistanche deserticola*; CP—*Castilleja paramensis*; CS—*Cistanche salsa*; CT—*Cistanche tubulosa*; DH—*Doroceras hygrometricum*; EG—*Erythranthe guttata*; HP—*Hesperelaea palmeri*; RS—*Rotheca serrate*; SM—*Salvai miltiorrhiza*; UR—*Utricularia reniformis*; Figure S23: Boxplots of pairwise Ka/Ks values in *nad*4L gene within 11 *Lamiales* species. AR—*Ajuga reptans*; CD—*Cistanche deserticola*; CP—*Castilleja paramensis*; CS—*Cistanche salsa*; CT—*Cistanche tubulosa*; DH—*Doroceras hygrometricum*; EG—*Erythranthe guttata*; HP—*Hesperelaea palmeri*; RS—*Rotheca serrate*; SM—*Salvai miltiorrhiza*; UR—*Utricularia reniformis*; Figure S24: Boxplots of pairwise Ka/Ks values in *nad*5 gene within 11 *Lamiales* species. AR—*Ajuga reptans*; CD—*Cistanche deserticola*; CP—*Castilleja paramensis*; CS—*Cistanche salsa*; CT—*Cistanche tubulosa*; DH—*Doroceras hygrometricum*; EG—*Erythranthe guttata*; HP—*Hesperelaea palmeri*; RS—*Rotheca serrate*; SM—*Salvai miltiorrhiza*; UR—*Utricularia reniformis*; Figure S25: Boxplots of pairwise Ka/Ks values in *nad*6 gene within 11 *Lamiales* species. AR—*Ajuga reptans*; CD—*Cistanche deserticola*; CP—*Castilleja paramensis*; CS—*Cistanche salsa*; CT—*Cistanche tubulosa*; DH—*Doroceras hygrometricum*; EG—*Erythranthe guttata*; HP—*Hesperelaea palmeri*; RS—*Rotheca serrate*; SM—*Salvai miltiorrhiza*; UR—*Utricularia reniformis*; Figure S26: Boxplots of pairwise Ka/Ks values in *nad*7 gene within 11 *Lamiales* species. AR—*Ajuga reptans*; CD—*Cistanche deserticola*; CP—*Castilleja paramensis*; CS—*Cistanche salsa*; CT—*Cistanche tubulosa*; DH—*Doroceras hygrometricum*; EG—*Erythranthe guttata*; HP—*Hesperelaea palmeri*; RS—*Rotheca serrate*; SM—*Salvai miltiorrhiza*; UR—*Utricularia reniformis*; Figure S27: Boxplots of pairwise Ka/Ks values in *rpl*5 gene within 11 *Lamiales* species. AR—*Ajuga reptans*; CD—*Cistanche deserticola*; CP—*Castilleja paramensis*; CS—*Cistanche salsa*; CT—*Cistanche tubulosa*; DH—*Doroceras hygrometricum*; EG—*Erythranthe guttata*; HP—*Hesperelaea palmeri*; RS—*Rotheca serrate*; SM—*Salvai miltiorrhiza*; UR—*Utricularia reniformis*; Figure S 28: Boxplots of pairwise Ka/Ks values in *rpl*10 gene within 11 *Lamiales* species. AR—*Ajuga reptans*; CD—*Cistanche deserticola*; CP—*Castilleja paramensis*; CS—*Cistanche salsa*; CT—*Cistanche tubulosa*; DH—*Doroceras hygrometricum*; EG—*Erythranthe guttata*; HP—*Hesperelaea palmeri*; RS—*Rotheca serrate*; SM—*Salvai miltiorrhiza*; UR—*Utricularia reniformis*; Figure S29: Boxplots of pairwise Ka/Ks values in *rps*12 gene within 11 *Lamiales* species. AR—*Ajuga reptans*; CD—*Cistanche deserticola*; CP—*Castilleja paramensis*; CS—*Cistanche salsa*; CT—*Cistanche tubulosa*; DH—*Doroceras hygrometricum*; EG—*Erythranthe guttata*; HP—*Hesperelaea palmeri*; RS—*Rotheca serrate*; SM—*Salvai miltiorrhiza*; UR—*Utricularia reniformis*; Figure S30: Boxplots of pairwise Ka/Ks values in *rps*13 gene within 11 *Lamiales* species. AR—*Ajuga reptans*; CD—*Cistanche deserticola*; CP—*Castilleja paramensis*; CS—*Cistanche salsa*; CT—*Cistanche tubulosa*; DH—*Doroceras hygrometricum*; EG—*Erythranthe guttata*; HP—*Hesperelaea palmeri*; RS—*Rotheca serrate*; SM—*Salvai miltiorrhiza*; UR—*Utricularia reniformis*; Figure S31: Boxplots of pairwise Ka/Ks values in *sdh*4 gene within 11 *Lamiales* species. AR—*Ajuga reptans*; CD—*Cistanche deserticola*; CP—*Castilleja paramensis*; CS—*Cistanche salsa*; CT—*Cistanche tubulosa*; DH—*Doroceras hygrometricum*; EG—*Erythranthe guttata*; HP—*Hesperelaea palmeri*; RS—*Rotheca serrate*; SM—*Salvai miltiorrhiza*; UR—*Utricularia reniformis*; Table S1: Sampling and sequencing data information; Table S2: Mitogenome information of 8 *Lamiales* species and 2 outgroups; Table S3: Mitochondrial genome information of Chenopodiaceae species used in HGT events prediction; Table S4: Comparison of mitochondrial protein coding genes copy with 11 *Lamiales* mitogenomes; Table S5: Mitogenome information of parasitic plants; Table S6: Comparison of mitochondrial protein coding genes copy with 9 parasitic plant mitogenomes; Table S7: List of potential MTPTs of *C. deserticola*; Table S8: List of potential MTPTs of *C. salsa*; Table S9: List of potential MTPTs of *C. tubulosa*; Table S10 Types and numbers of SSRs in the *Cistanche* mitogenome; Table S11: Interspersed repeat sequences identified in *C. deserticola* mitogenome; Table S12: Interspersed repeat sequences identified in *C. salsa* mitogenome; Table S13: Interspersed repeat sequences identified in *C. tubulosa* mitogenome; Table S14: Tandem repeat sequences identified in the mitogenome of *C. deserticola*;Table S15: Tandem repeat sequences identified in the mitogenome of *C. salsa*; Table S16: Tandem repeat sequences identified in the mitogenome of *C. tubulosa*; Table S17: Segment duplication sequences in *C. deserticola* mitogenome; Table S18: Segment duplication sequences in *C. salsa* mitogenome; Table S19: Segment duplication sequences in *C. tubulosa* mitogenome; Table S20: Pairwise Ka/Ks ratios in different mitochondrial genes of 11 *Lamiales* plants.
