# Supplementary material for: Cistanche Species Mitogenomes Suggest Diversity and Complexity in Lamiales-Order Mitogenomes
Source: Genes (Basel). 2022 Oct 4;13(10):1791. doi: 10.3390/genes13101791 (PMC9602076; doi:10.3390/genes13101791)
Supplement: Supplementary file 1 [file genes-13-01791-s001.zip › 2022-09-07Supplementary Figures.pdf]

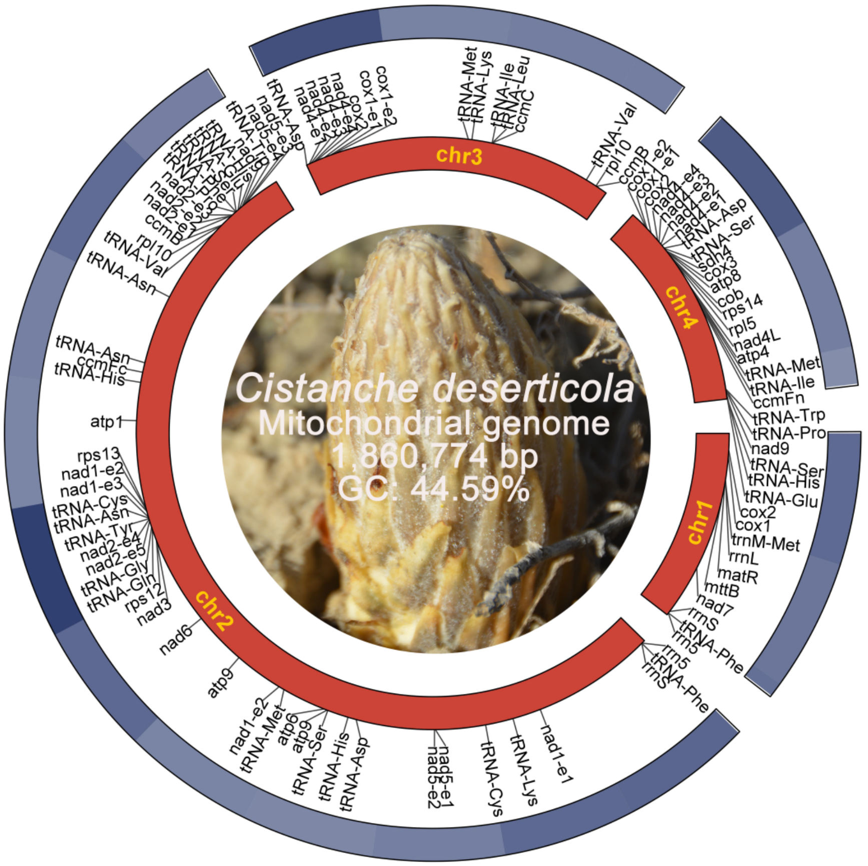

FIGURE S1 | Mitochondrial genome map of *Cistanche deserticola*. The outside circle shows the GC content. The inside circle represents the protein coding gene, tRNA and rRNA.

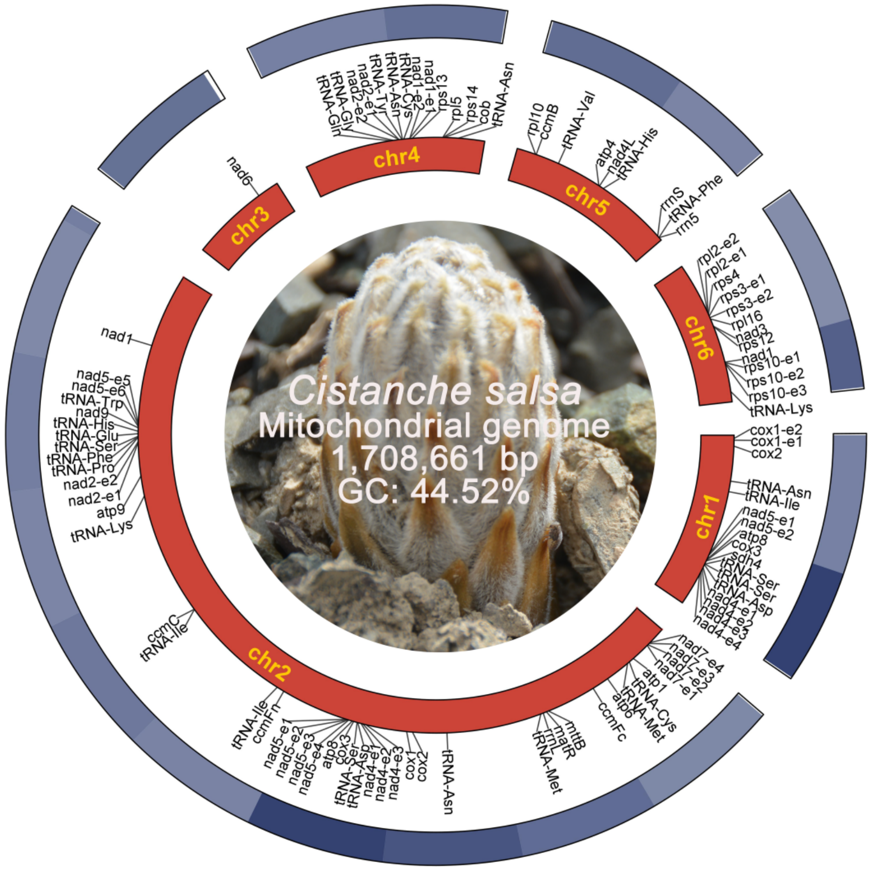

FIGURE S2 | Mitochondrial genome map of *Cistanche salsa*. The outside circle shows the GC content. The inside circle represents the protein coding gene, tRNA and rRNA.

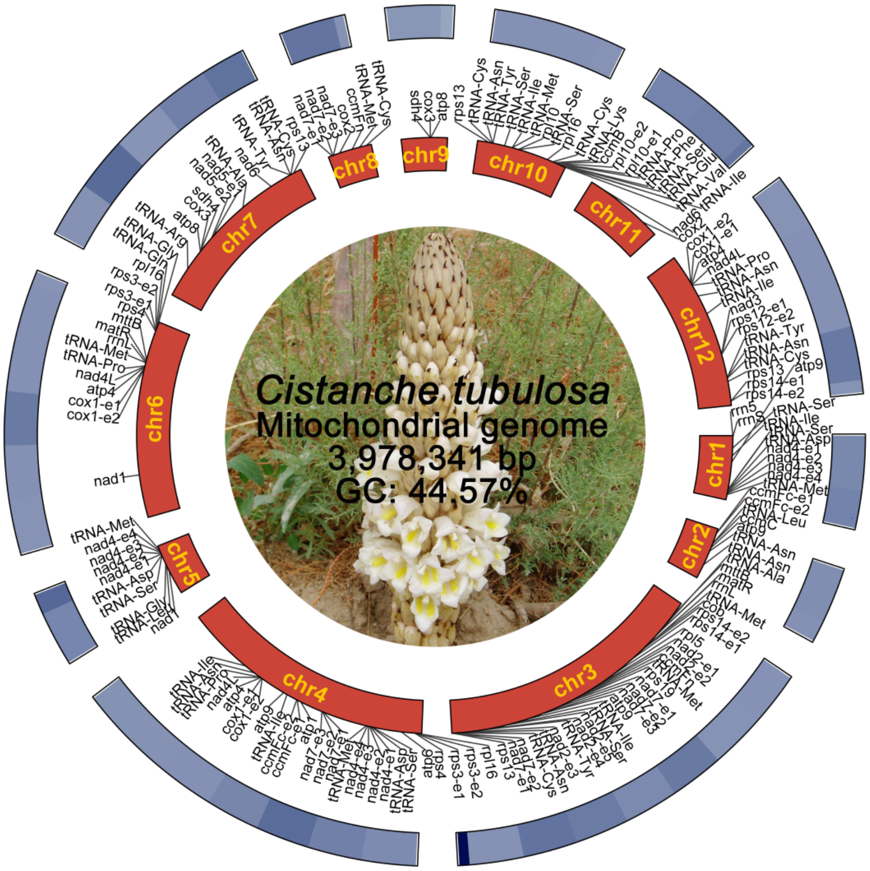

FIGURE S3 | Mitochondrial genome map of *Cistanche tubulosa*. The outside circle shows the GC content. The inside circle represents the protein coding gene, tRNA and rRNA.

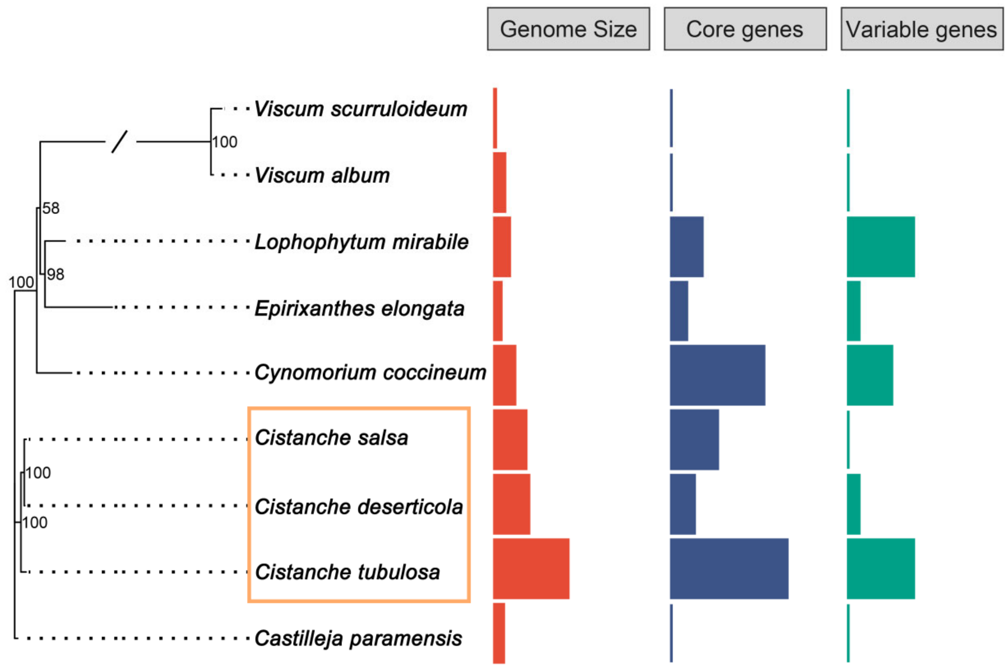

FIGURE S4 | Genome size and protein-coding gene content of 9 parasitic plants mitochondrial genomes.

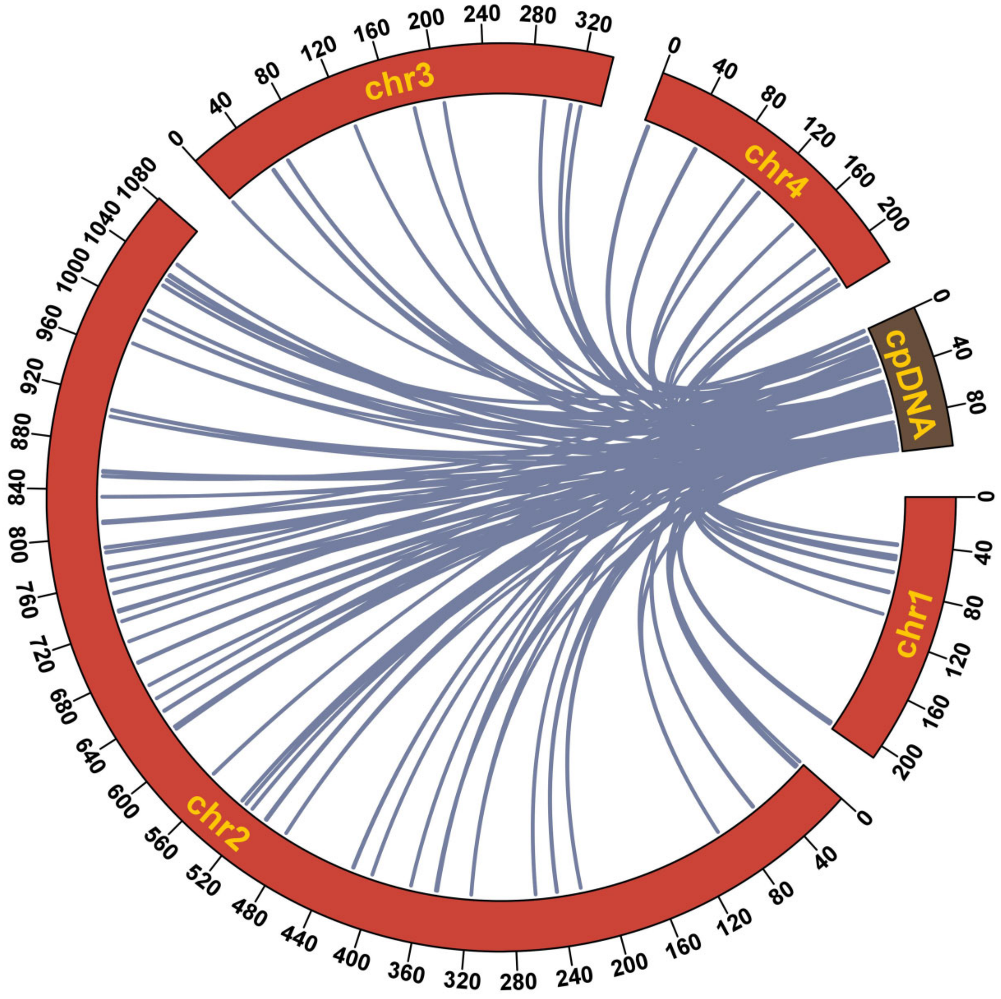

FIGURE S5 | The collinearity analysis among *C. deserticola* mitochondrial genome and plastid genome.

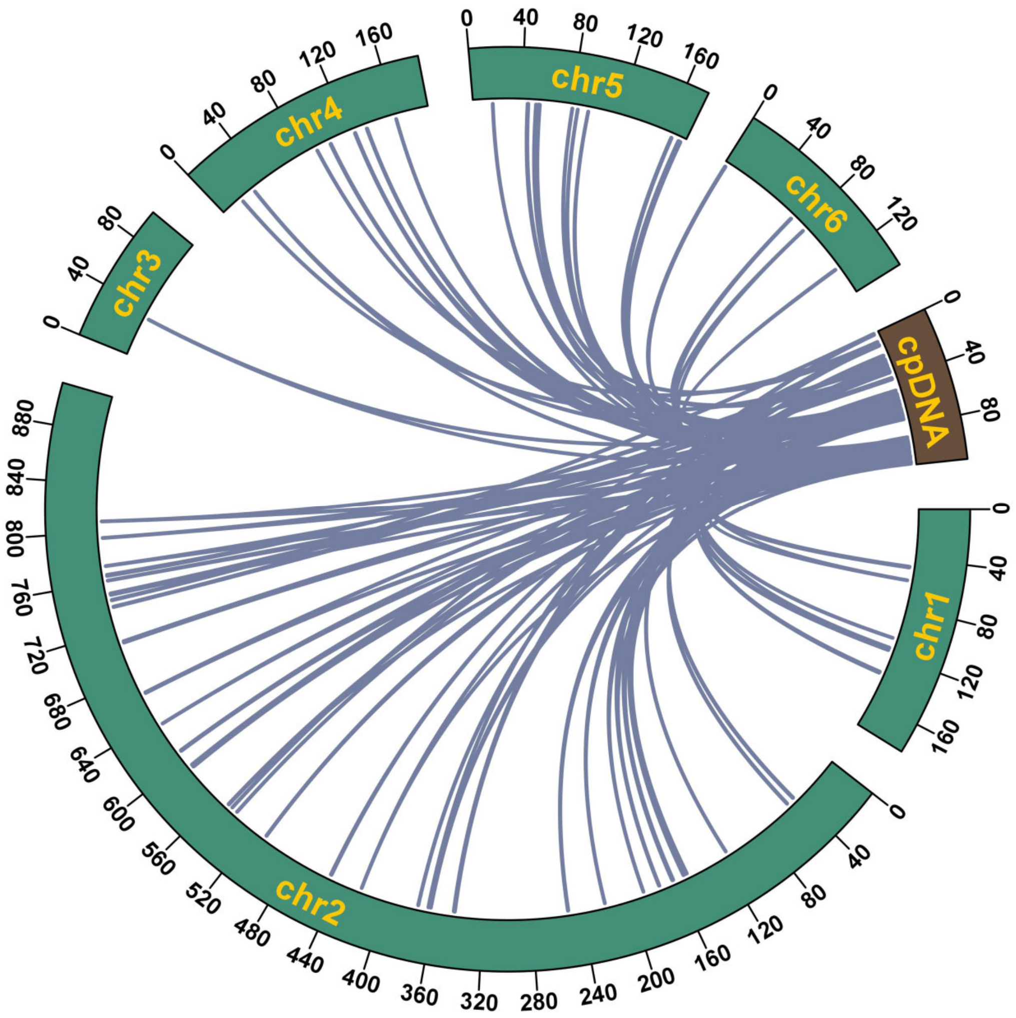

FIGURE S6 | The collinearity analysis among *C. salsa* mitochondrial genome and plastid genome.

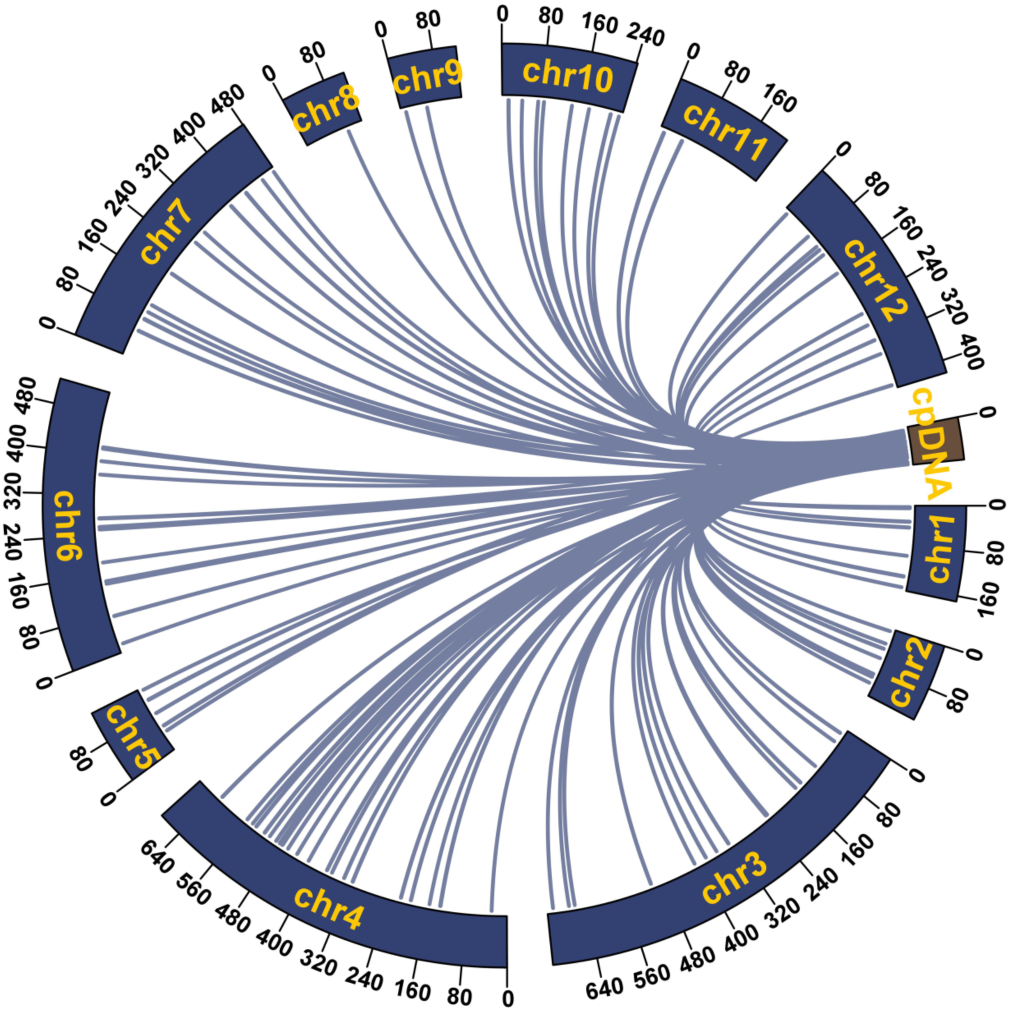

FIGURE S7 | The collinearity analysis among *C. tubulosa* mitochondrial genome and plastid genome.

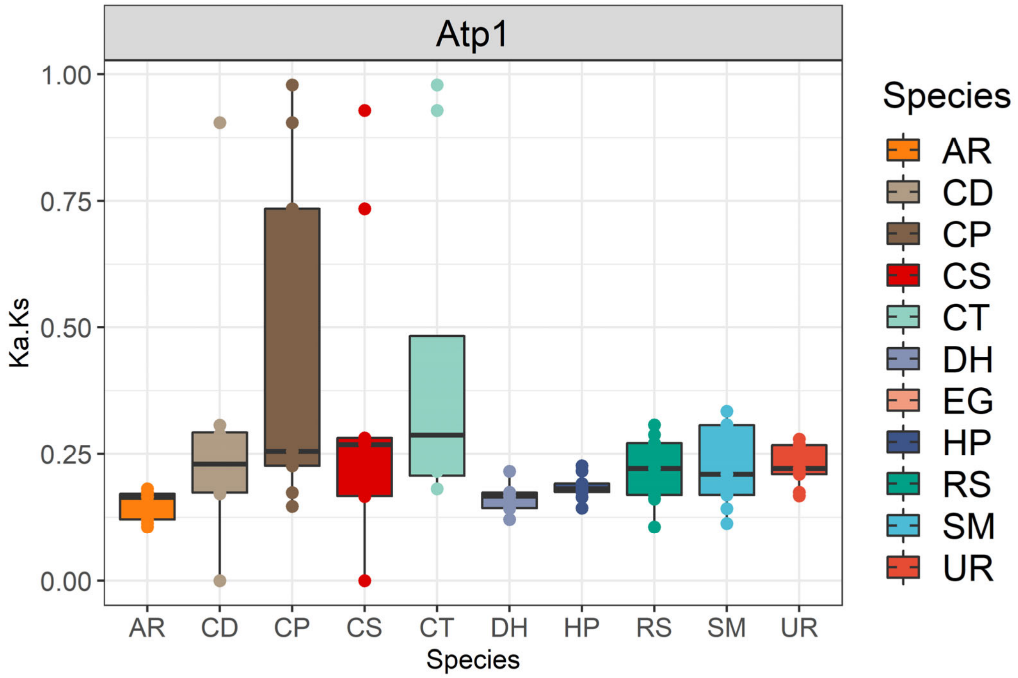

FIGURE S8 | Boxplots of pairwise Ka/Ks values in *Atp*1 gene within 11 Lamiales species. AR – *Ajuga reptans*; CD – *Cistanche deserticola*; CP – *Castilleja paramensis*; CS – *Cistanche salsa*; CT – *Cistanche tubulosa*; DH – *Doroceras hygrometricum*; EG – *Erythranthe guttata*; HP – *Hesperelaea palmeri*; RS – *Rotheca serrate*; SM – *Salvai miltiorrhiza*; UR – *Utricularia reniformis*.

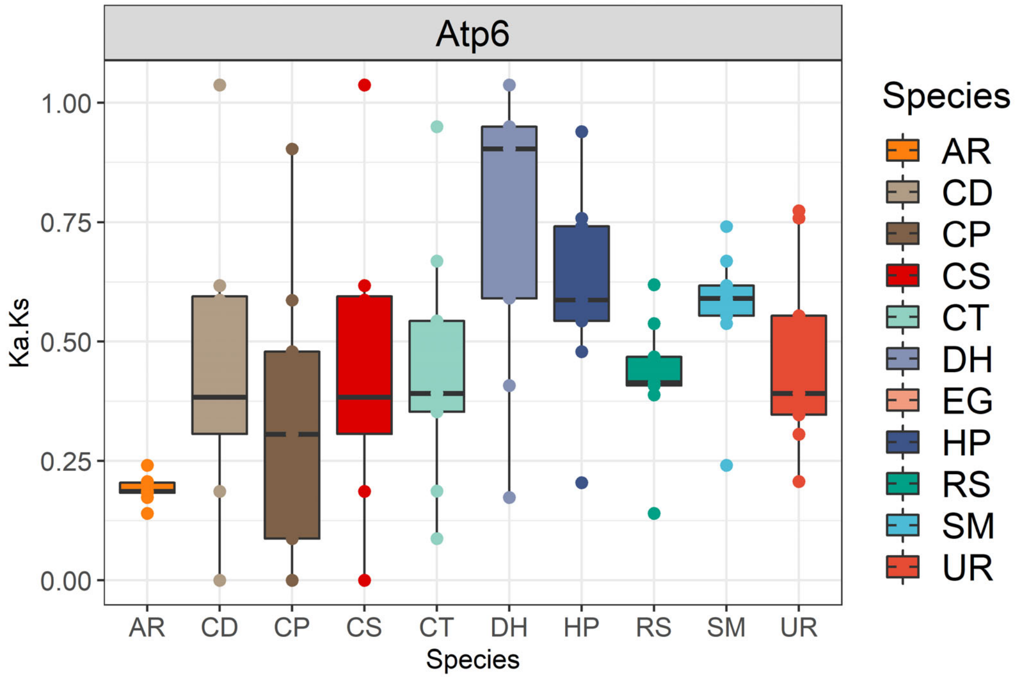

FIGURE S9 | Boxplots of pairwise Ka/Ks values in *Atp*6 gene within 11 Lamiales species. AR – *Ajuga reptans*; CD – *Cistanche deserticola*; CP – *Castilleja paramensis*; CS – *Cistanche salsa*; CT – *Cistanche tubulosa*; DH – *Doroceras hygrometricum*; EG – *Erythranthe guttata*; HP – *Hesperelaea palmeri*; RS – *Rotheca serrate*; SM – *Salvai miltiorrhiza*; UR – *Utricularia reniformis*.

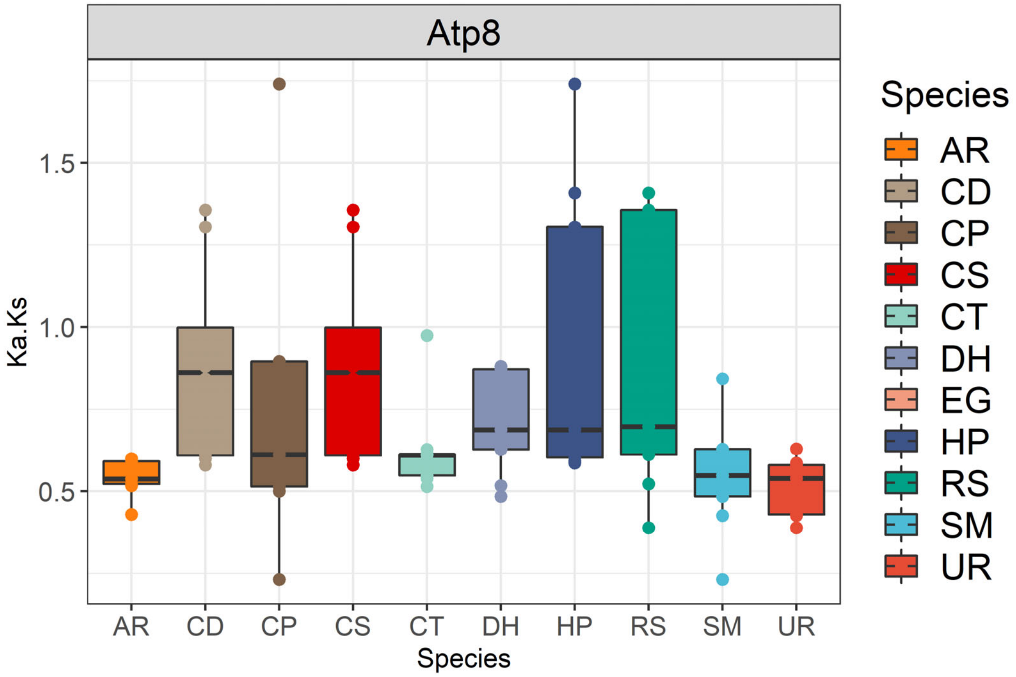

FIGURE S10 | Boxplots of pairwise Ka/Ks values in *Atp*8 gene within 11 Lamiales species. AR – *Ajuga reptans*; CD – *Cistanche deserticola*; CP – *Castilleja paramensis*; CS – *Cistanche salsa*; CT – *Cistanche tubulosa*; DH – *Doroceras hygrometricum*; EG – *Erythranthe guttata*; HP – *Hesperelaea palmeri*; RS – *Rotheca serrate*; SM – *Salvai miltiorrhiza*; UR – *Utricularia reniformis*.

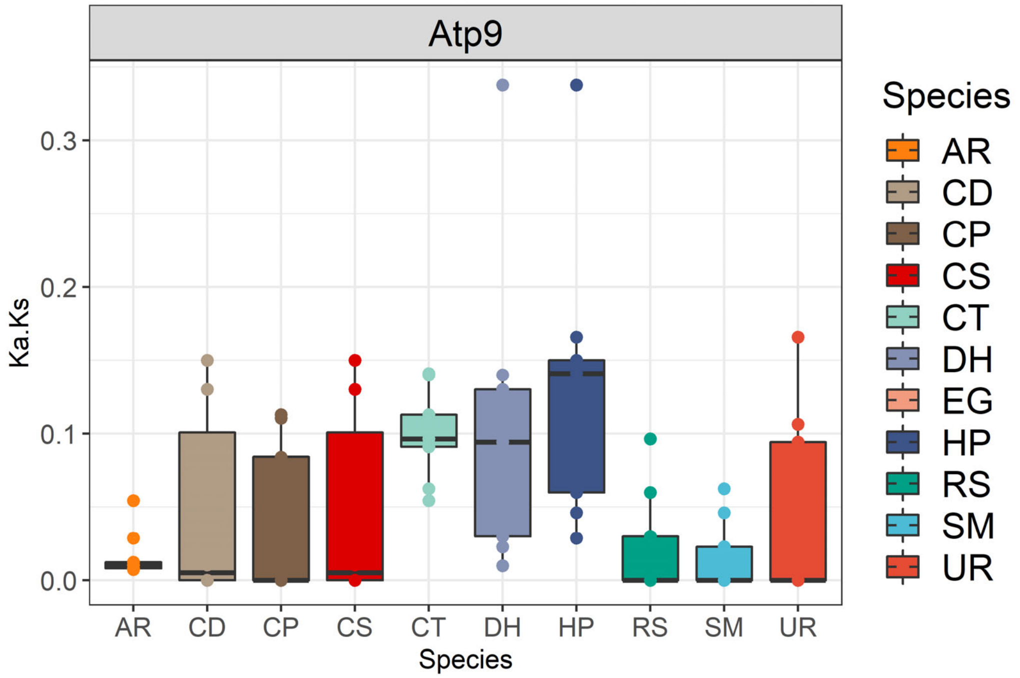

FIGURE S11 | Boxplots of pairwise Ka/Ks values in *Atp*9 gene within 11 Lamiales species. AR – *Ajuga reptans*; CD – *Cistanche deserticola*; CP – *Castilleja paramensis*; CS – *Cistanche salsa*; CT – *Cistanche tubulosa*; DH – *Doroceras hygrometricum*; EG – *Erythranthe guttata*; HP – *Hesperelaea palmeri*; RS – *Rotheca serrate*; SM – *Salvai miltiorrhiza*; UR – *Utricularia reniformis*.

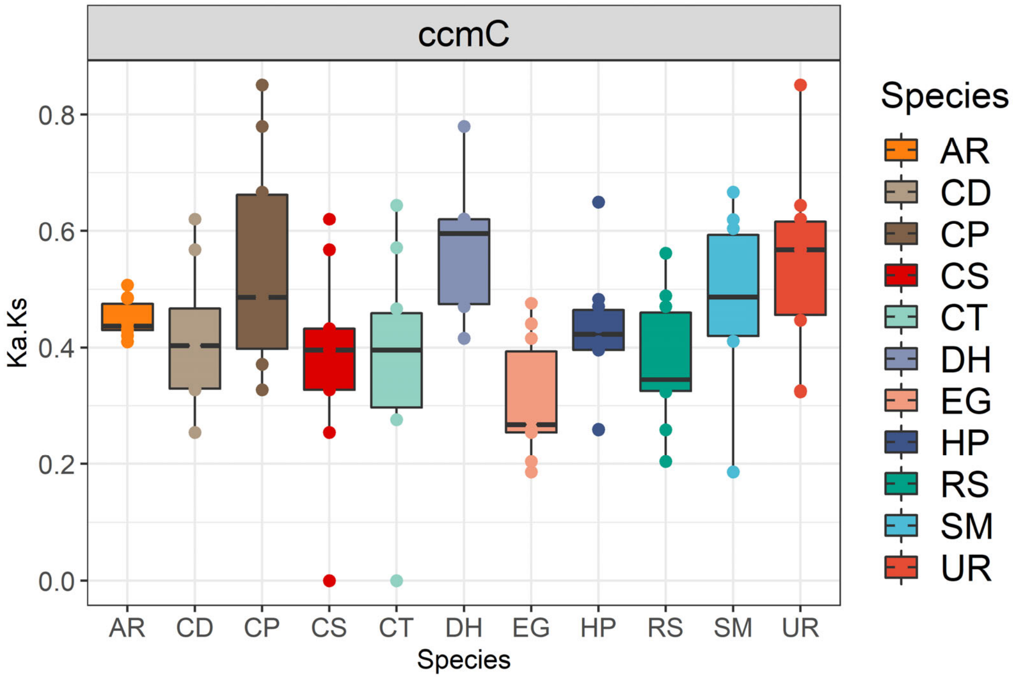

FIGURE S12 | Boxplots of pairwise Ka/Ks values in *ccmC* gene within 11 Lamiales species. AR – *Ajuga reptans*; CD – *Cistanche deserticola*; CP – *Castilleja paramensis*; CS – *Cistanche salsa*; CT – *Cistanche tubulosa*; DH – *Doroceras hygrometricum*; EG – *Erythranthe guttata*; HP – *Hesperelaea palmeri*; RS – *Rotheca serrate*; SM – *Salvai miltiorrhiza*; UR – *Utricularia reniformis*.

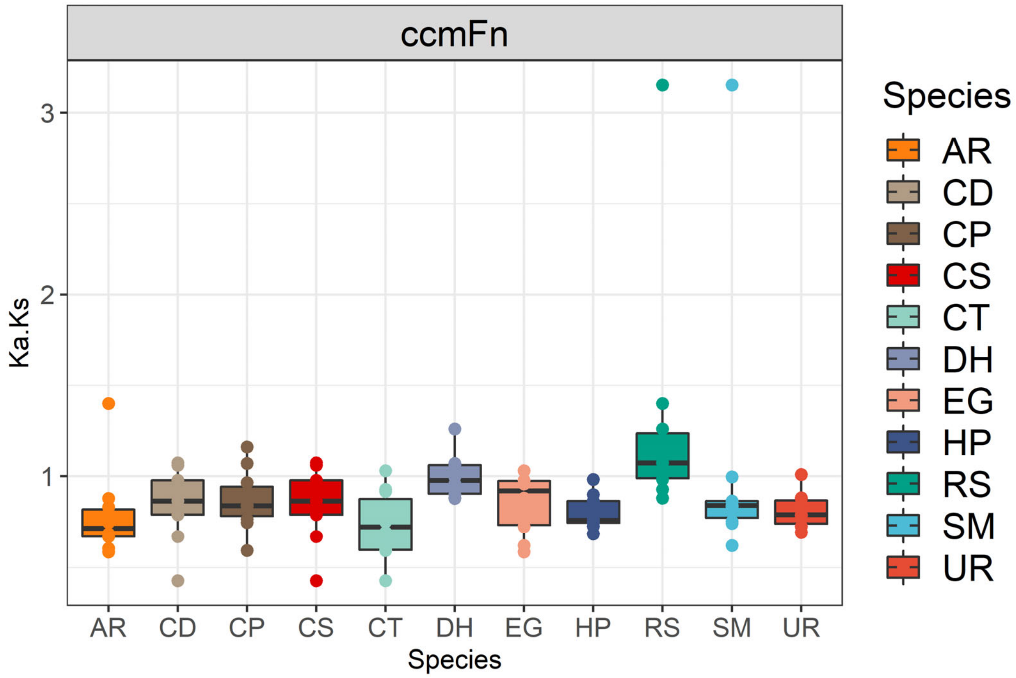

FIGURE S13 | Boxplots of pairwise Ka/Ks values in *ccmFn* gene within 11 Lamiales species. AR – *Ajuga reptans*; CD – *Cistanche deserticola*; CP – *Castilleja paramensis*; CS – *Cistanche salsa*; CT – *Cistanche tubulosa*; DH – *Doroceras hygrometricum*; EG – *Erythranthe guttata*; HP – *Hesperelaea palmeri*; RS – *Rotheca serrate*; SM – *Salvai miltiorrhiza*; UR – *Utricularia reniformis*.

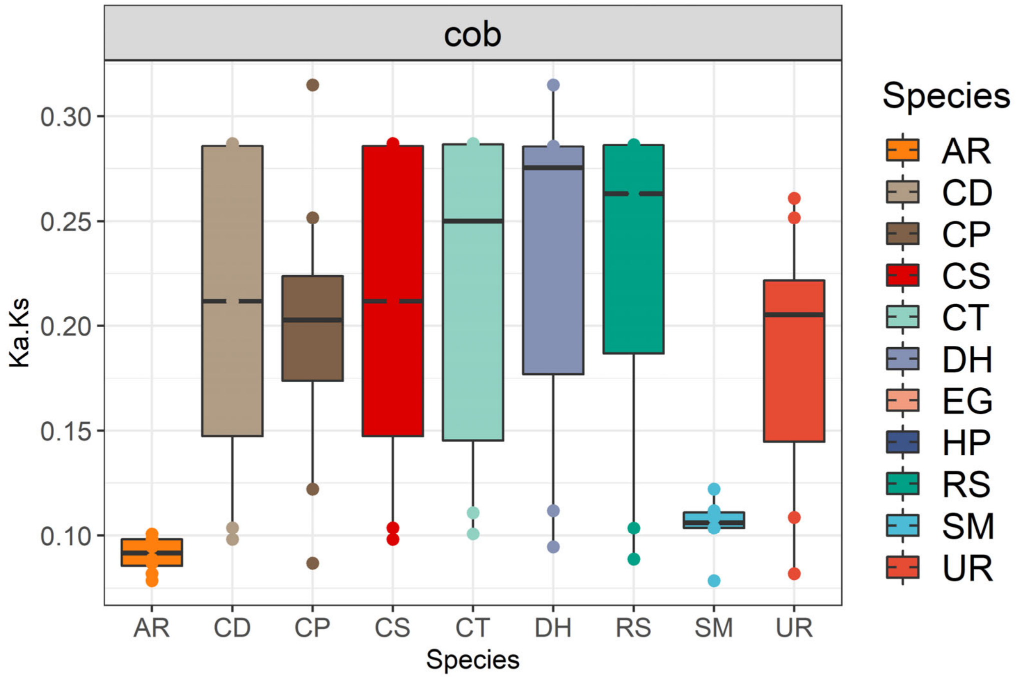

FIGURE S14 | Boxplots of pairwise Ka/Ks values in *cob* gene within 11 Lamiales species. AR – *Ajuga reptans*; CD – *Cistanche deserticola*; CP – *Castilleja paramensis*; CS – *Cistanche salsa*; CT – *Cistanche tubulosa*; DH – *Doroceras hygrometricum*; EG – *Erythranthe guttata*; HP – *Hesperelaea palmeri*; RS – *Rotheca serrate*; SM – *Salvai miltiorrhiza*; UR – *Utricularia reniformis*.

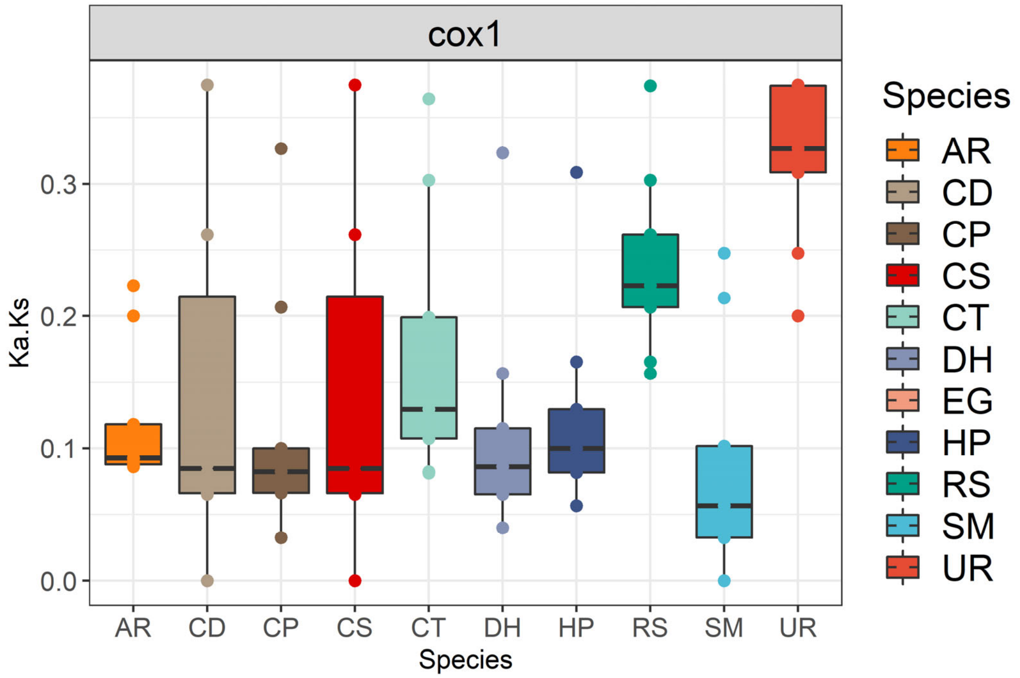

FIGURE S15 | Boxplots of pairwise Ka/Ks values in *cox*1 gene within 11 Lamiales species. AR – *Ajuga reptans*; CD – *Cistanche deserticola*; CP – *Castilleja paramensis*; CS – *Cistanche salsa*; CT – *Cistanche tubulosa*; DH – *Doroceras hygrometricum*; EG – *Erythranthe guttata*; HP – *Hesperelaea palmeri*; RS – *Rotheca serrate*; SM – *Salvai miltiorrhiza*; UR – *Utricularia reniformis*.

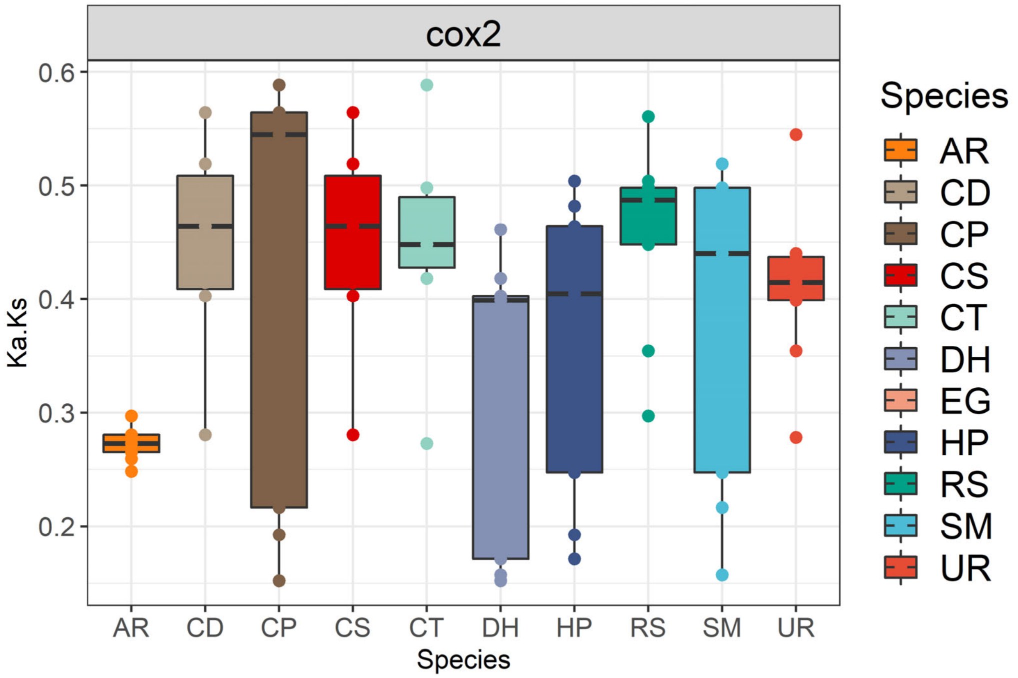

FIGURE S16 | Boxplots of pairwise Ka/Ks values in *cox*2 gene within 11 Lamiales species. AR – *Ajuga reptans*; CD – *Cistanche deserticola*; CP – *Castilleja paramensis*; CS – *Cistanche salsa*; CT – *Cistanche tubulosa*; DH – *Doroceras hygrometricum*; EG – *Erythranthe guttata*; HP – *Hesperelaea palmeri*; RS – *Rotheca serrate*; SM – *Salvai miltiorrhiza*; UR – *Utricularia reniformis*.

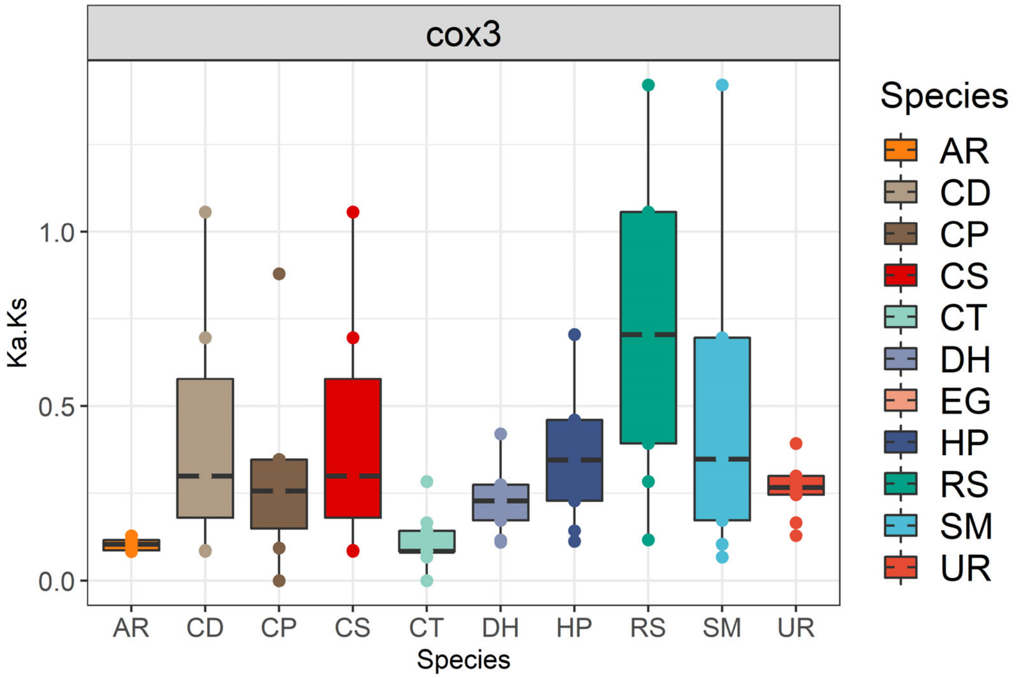

FIGURE S17 | Boxplots of pairwise Ka/Ks values in *cox*3 gene within 11 Lamiales species. AR – *Ajuga reptans*; CD – *Cistanche deserticola*; CP – *Castilleja paramensis*; CS – *Cistanche salsa*; CT – *Cistanche tubulosa*; DH – *Doroceras hygrometricum*; EG – *Erythranthe guttata*; HP – *Hesperelaea palmeri*; RS – *Rotheca serrate*; SM – *Salvai miltiorrhiza*; UR – *Utricularia reniformis*.

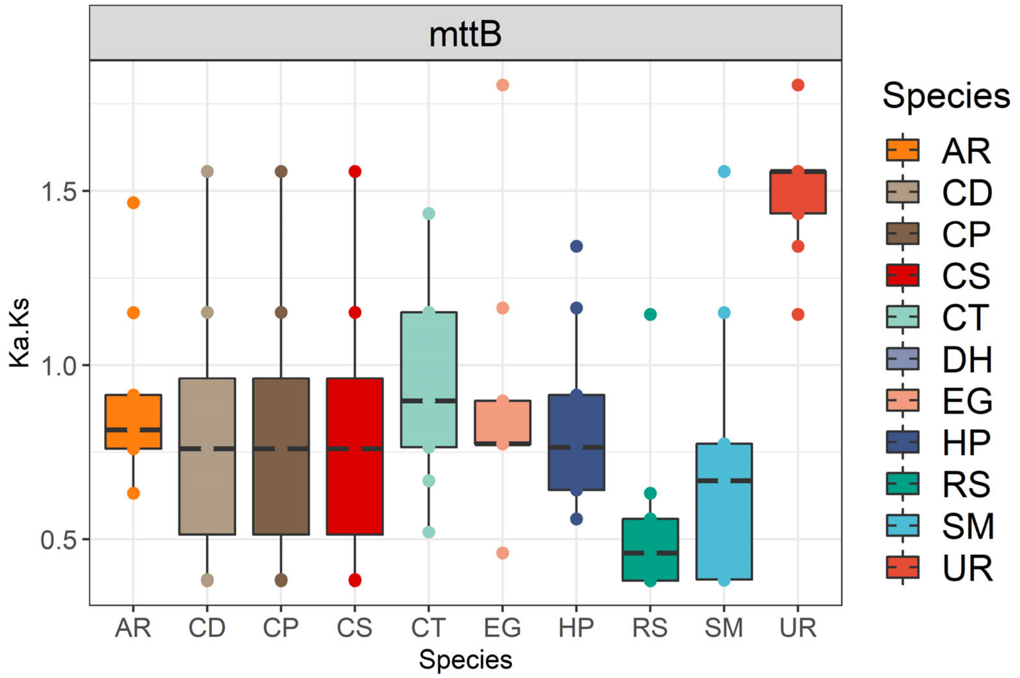

FIGURE S18 | Boxplots of pairwise Ka/Ks values in *mttB* gene within 11 Lamiales species. AR – *Ajuga reptans*; CD – *Cistanche deserticola*; CP – *Castilleja paramensis*; CS – *Cistanche salsa*; CT – *Cistanche tubulosa*; DH – *Doroceras hygrometricum*; EG – *Erythranthe guttata*; HP – *Hesperelaea palmeri*; RS – *Rotheca serrate*; SM – *Salvai miltiorrhiza*; UR – *Utricularia reniformis*.

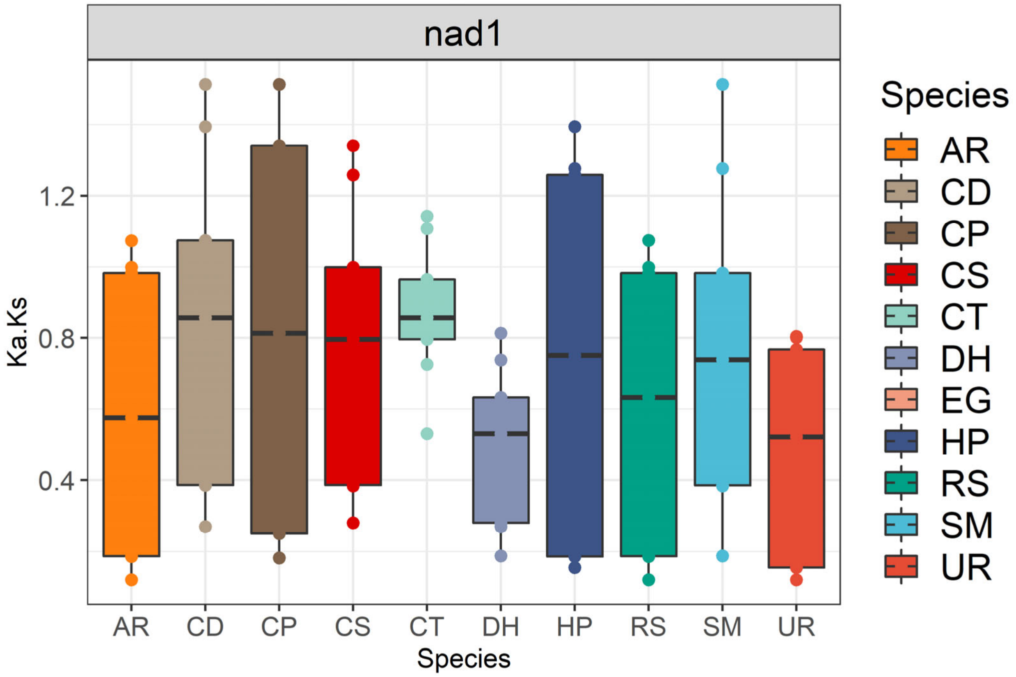

FIGURE S19 | Boxplots of pairwise Ka/Ks values in *nad*1 gene within 11 Lamiales species. AR – *Ajuga reptans*; CD – *Cistanche deserticola*; CP – *Castilleja paramensis*; CS – *Cistanche salsa*; CT – *Cistanche tubulosa*; DH – *Doroceras hygrometricum*; EG – *Erythranthe guttata*; HP – *Hesperelaea palmeri*; RS – *Rotheca serrate*; SM – *Salvai miltiorrhiza*; UR – *Utricularia reniformis*.

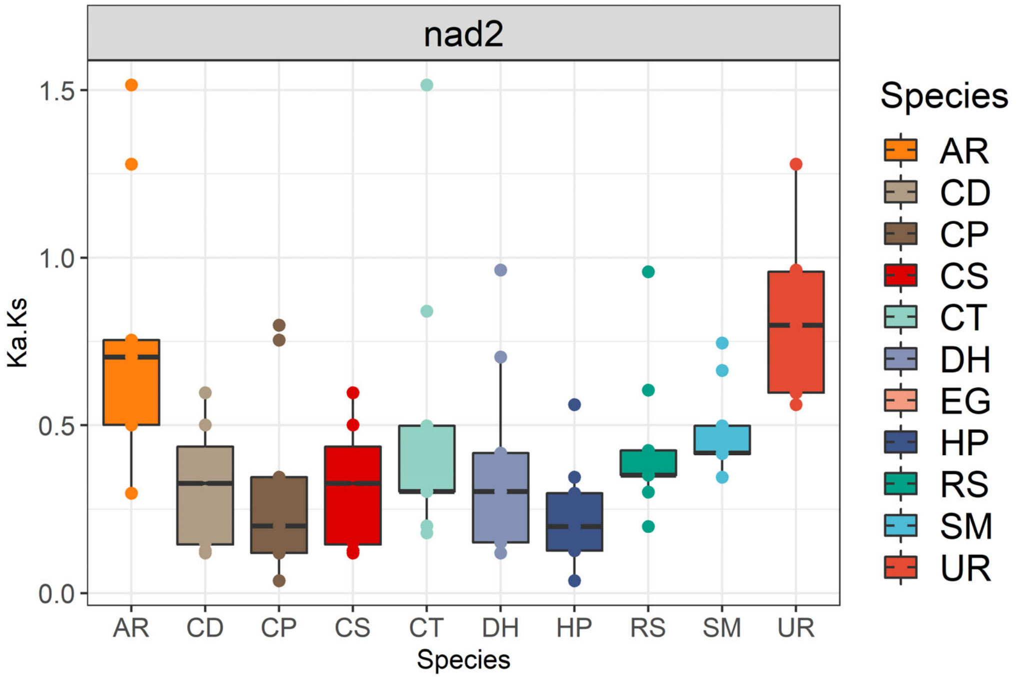

FIGURE S20 | Boxplots of pairwise Ka/Ks values in *nad*2 gene within 11 Lamiales species. AR – *Ajuga reptans*; CD – *Cistanche deserticola*; CP – *Castilleja paramensis*; CS – *Cistanche salsa*; CT – *Cistanche tubulosa*; DH – *Doroceras hygrometricum*; EG – *Erythranthe guttata*; HP – *Hesperelaea palmeri*; RS – *Rotheca serrate*; SM – *Salvai miltiorrhiza*; UR – *Utricularia reniformis*.

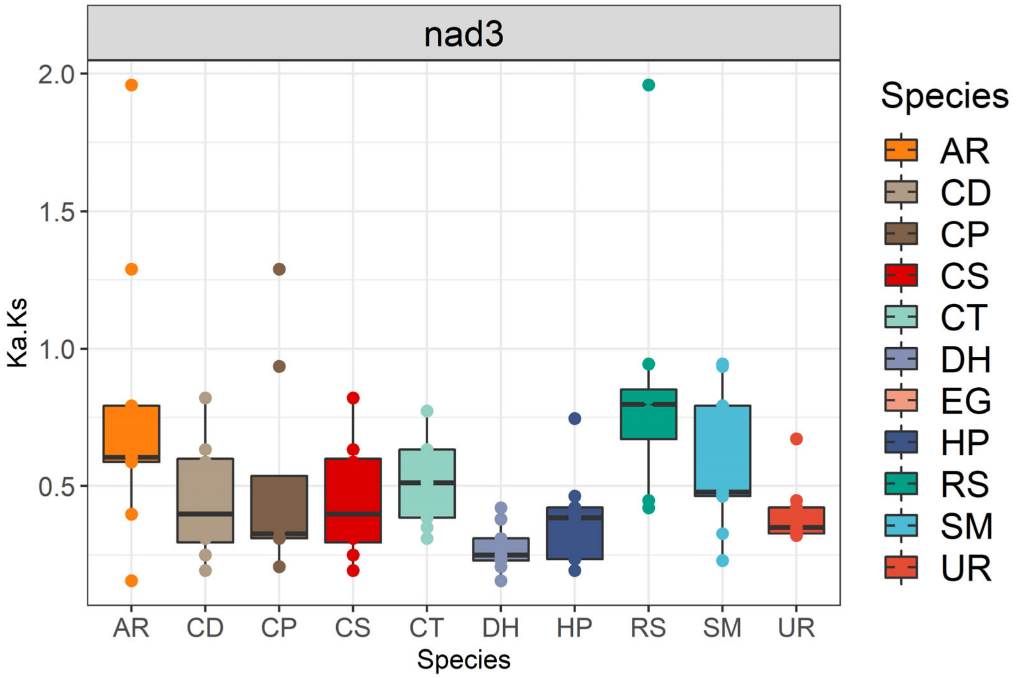

FIGURE S21 | Boxplots of pairwise Ka/Ks values in *nad*3 gene within 11 Lamiales species. AR – *Ajuga reptans*; CD – *Cistanche deserticola*; CP – *Castilleja paramensis*; CS – *Cistanche salsa*; CT – *Cistanche tubulosa*; DH – *Doroceras hygrometricum*; EG – *Erythranthe guttata*; HP – *Hesperelaea palmeri*; RS – *Rotheca serrate*; SM – *Salvai miltiorrhiza*; UR – *Utricularia reniformis*.

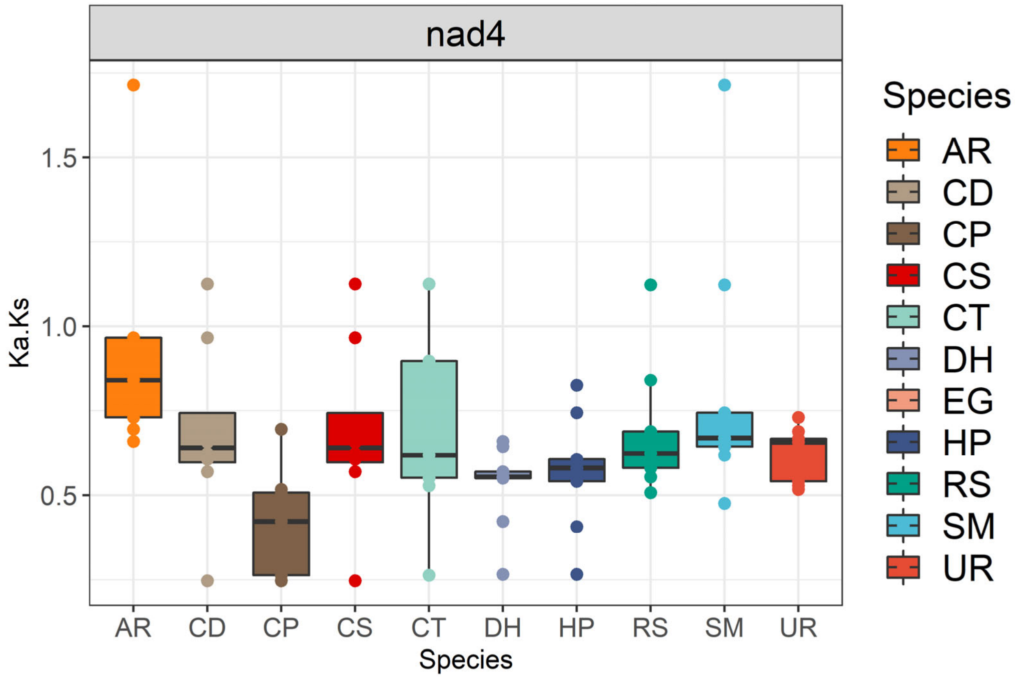

FIGURE S22 | Boxplots of pairwise Ka/Ks values in *nad*4 gene within 11 Lamiales species. AR – *Ajuga reptans*; CD – *Cistanche deserticola*; CP – *Castilleja paramensis*; CS – *Cistanche salsa*; CT – *Cistanche tubulosa*; DH – *Doroceras hygrometricum*; EG – *Erythranthe guttata*; HP – *Hesperelaea palmeri*; RS – *Rotheca serrate*; SM – *Salvai miltiorrhiza*; UR – *Utricularia reniformis*.

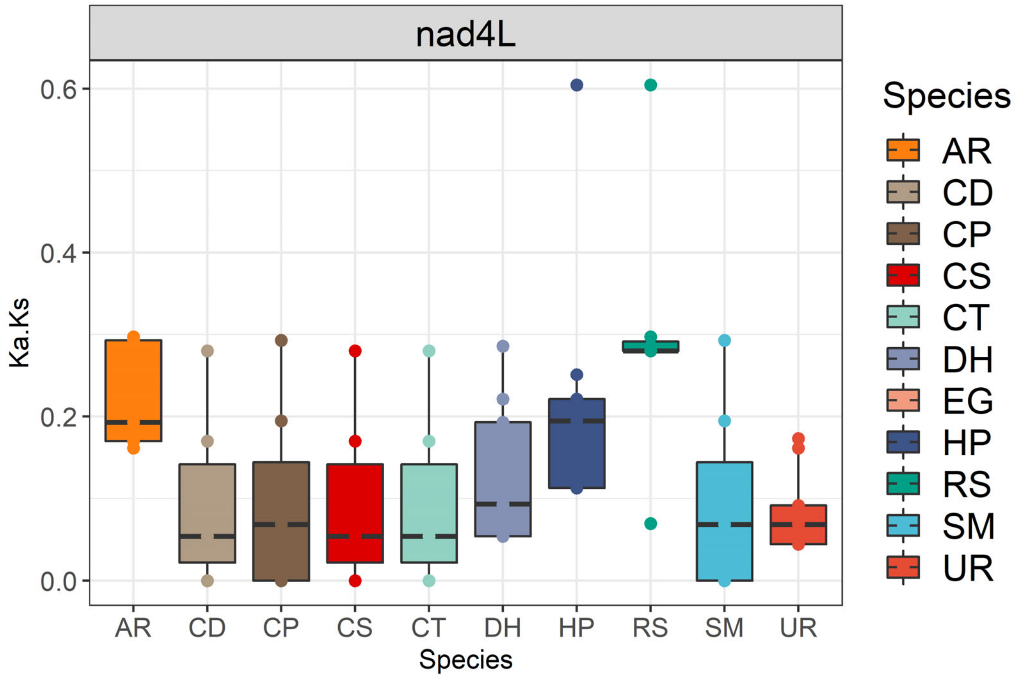

FIGURE S23 | Boxplots of pairwise Ka/Ks values in *nad*4L gene within 11 Lamiales species. AR – *Ajuga reptans*; CD – *Cistanche deserticola*; CP – *Castilleja paramensis*; CS – *Cistanche salsa*; CT – *Cistanche tubulosa*; DH – *Doroceras hygrometricum*; EG – *Erythranthe guttata*; HP – *Hesperelaea palmeri*; RS – *Rotheca serrate*; SM – *Salvai miltiorrhiza*; UR – *Utricularia reniformis*.

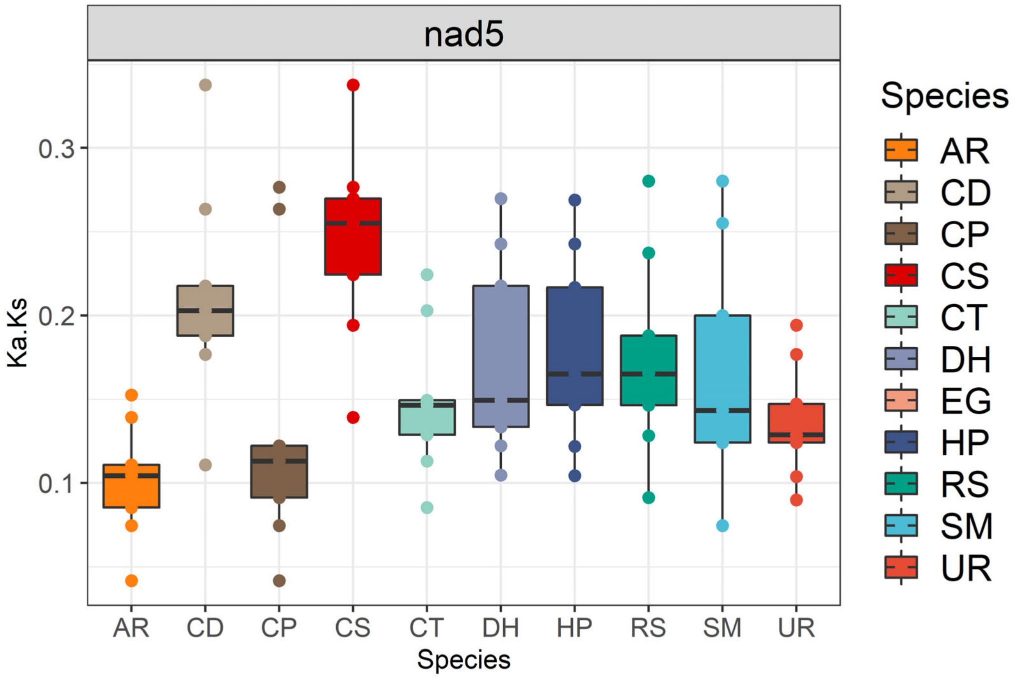

FIGURE S24 | Boxplots of pairwise Ka/Ks values in *nad*5 gene within 11 Lamiales species. AR – *Ajuga reptans*; CD – *Cistanche deserticola*; CP – *Castilleja paramensis*; CS – *Cistanche salsa*; CT – *Cistanche tubulosa*; DH – *Doroceras hygrometricum*; EG – *Erythranthe guttata*; HP – *Hesperelaea palmeri*; RS – *Rotheca serrate*; SM – *Salvai miltiorrhiza*; UR – *Utricularia reniformis*.

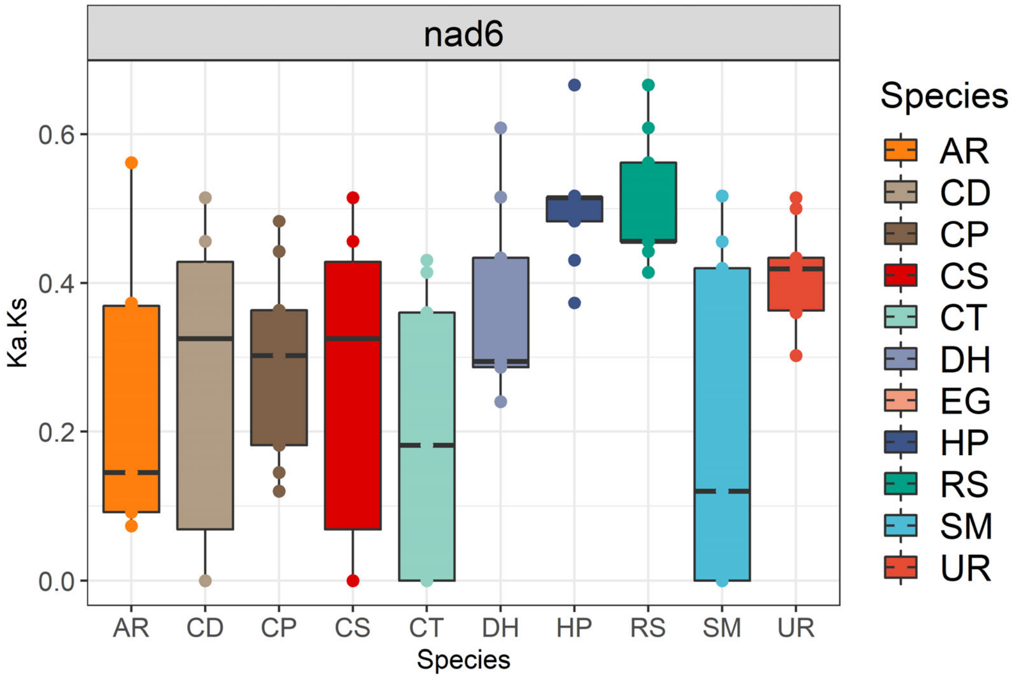

FIGURE S25 | Boxplots of pairwise Ka/Ks values in *nad6* gene within 11 Lamiales species. AR – *Ajuga reptans*; CD – *Cistanche deserticola*; CP – *Castilleja paramensis*; CS – *Cistanche salsa*; CT – *Cistanche tubulosa*; DH – *Doroceras hygrometricum*; EG – *Erythranthe guttata*; HP – *Hesperelaea palmeri*; RS – *Rotheca serrate*; SM – *Salvai miltiorrhiza*; UR – *Utricularia reniformis*.

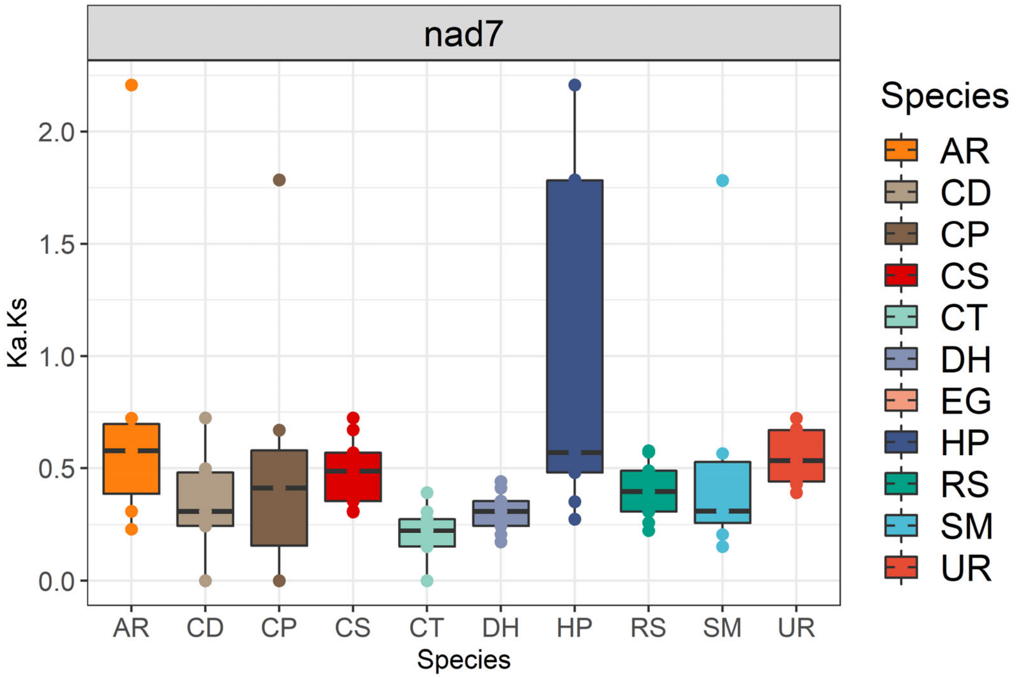

FIGURE S26 | Boxplots of pairwise Ka/Ks values in *nad7* gene within 11 Lamiales species. AR – *Ajuga reptans*; CD – *Cistanche deserticola*; CP – *Castilleja paramensis*; CS – *Cistanche salsa*; CT – *Cistanche tubulosa*; DH – *Doroceras hygrometricum*; EG – *Erythranthe guttata*; HP – *Hesperelaea palmeri*; RS – *Rotheca serrate*; SM – *Salvai miltiorrhiza*; UR – *Utricularia reniformis*.

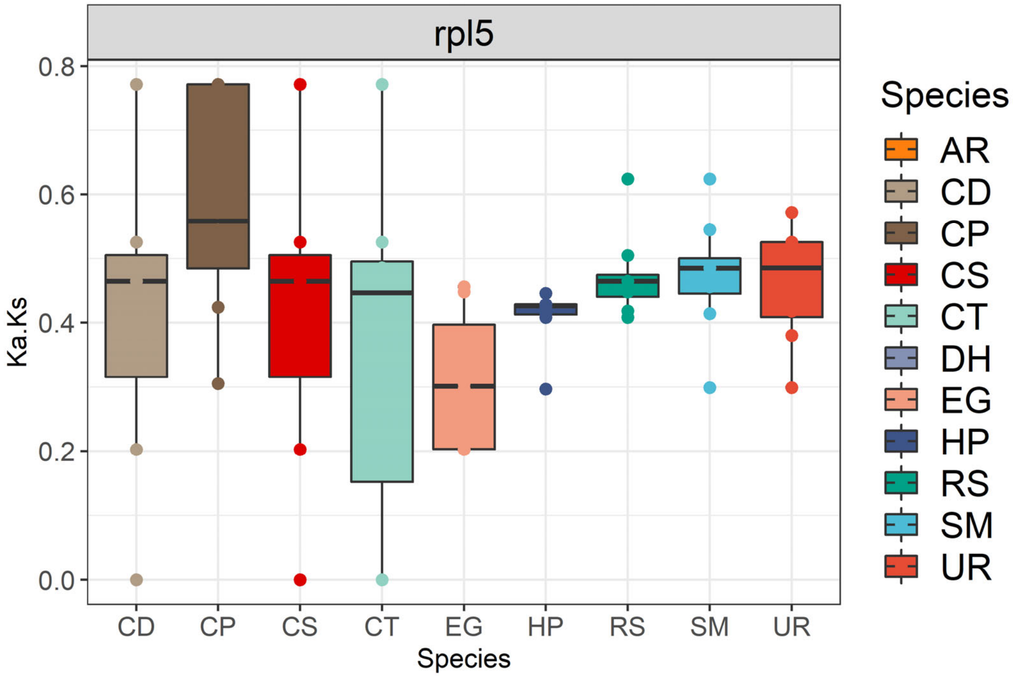

FIGURE S27 | Boxplots of pairwise Ka/Ks values in *rpl*5 gene within 11 Lamiales species. AR – *Ajuga reptans*; CD – *Cistanche deserticola*; CP – *Castilleja paramensis*; CS – *Cistanche salsa*; CT – *Cistanche tubulosa*; DH – *Doroceras hygrometricum*; EG – *Erythranthe guttata*; HP – *Hesperelaea palmeri*; RS – *Rotheca serrate*; SM – *Salvai miltiorrhiza*; UR – *Utricularia reniformis*.

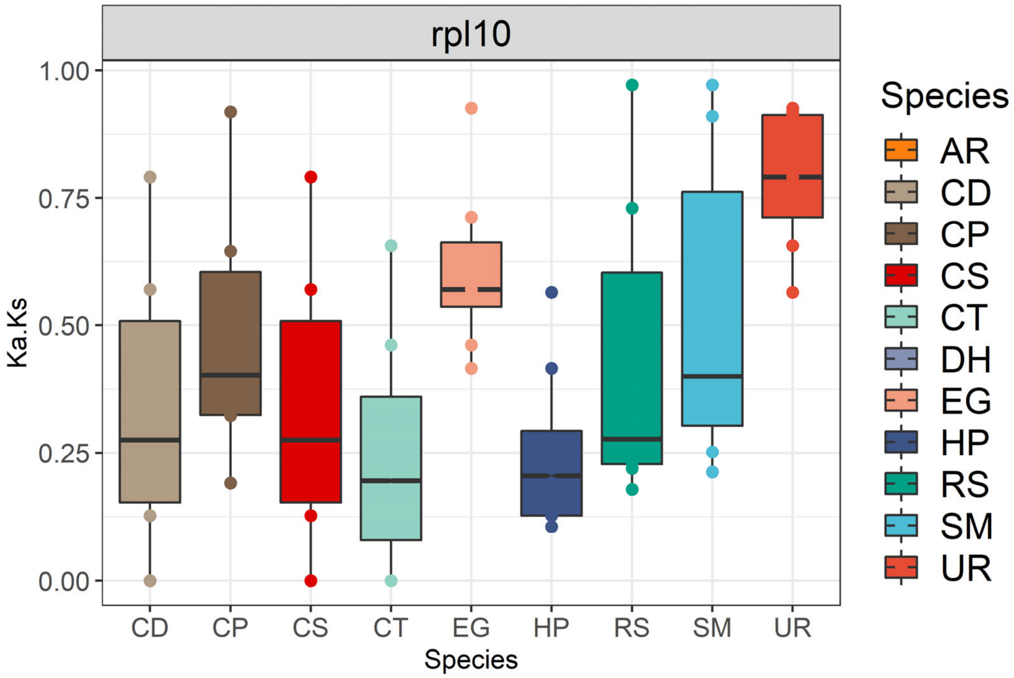

FIGURE S28 | Boxplots of pairwise Ka/Ks values in *rpl*10 gene within 11 Lamiales species. AR – *Ajuga reptans*; CD – *Cistanche deserticola*; CP – *Castilleja paramensis*; CS – *Cistanche salsa*; CT – *Cistanche tubulosa*; DH – *Doroceras hygrometricum*; EG – *Erythranthe guttata*; HP – *Hesperelaea palmeri*; RS – *Rotheca serrate*; SM – *Salvai miltiorrhiza*; UR – *Utricularia reniformis*.

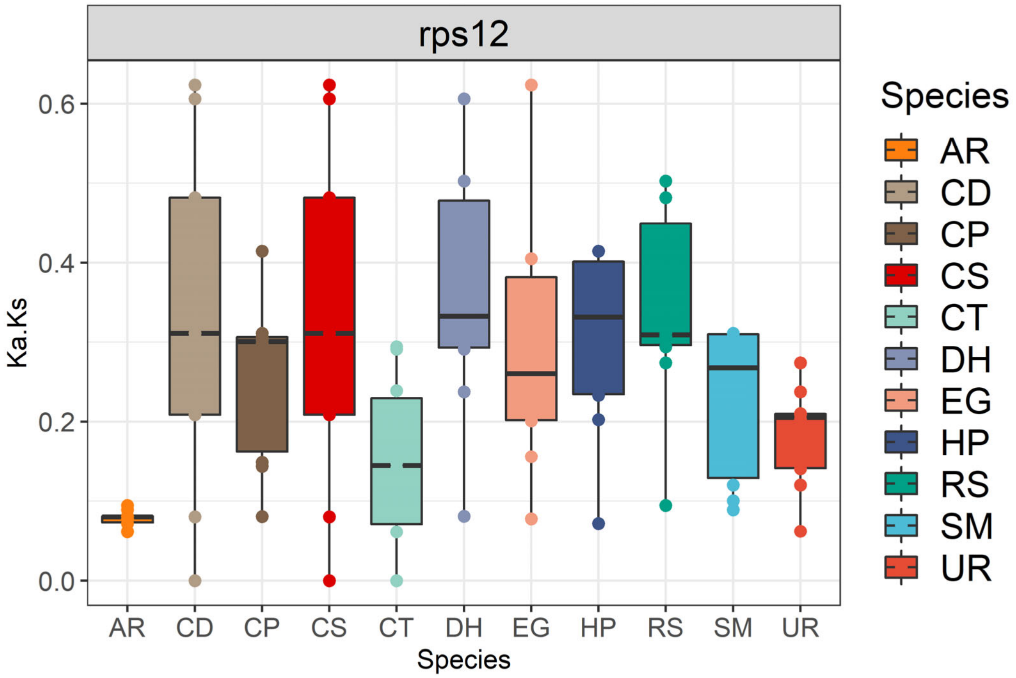

FIGURE S29 | Boxplots of pairwise Ka/Ks values in *rps*12 gene within 11 Lamiales species. AR – *Ajuga reptans*; CD – *Cistanche deserticola*; CP – *Castilleja paramensis*; CS – *Cistanche salsa*; CT – *Cistanche tubulosa*; DH – *Doroceras hygrometricum*; EG – *Erythranthe guttata*; HP – *Hesperelaea palmeri*; RS – *Rotheca serrate*; SM – *Salvai miltiorrhiza*; UR – *Utricularia reniformis*.

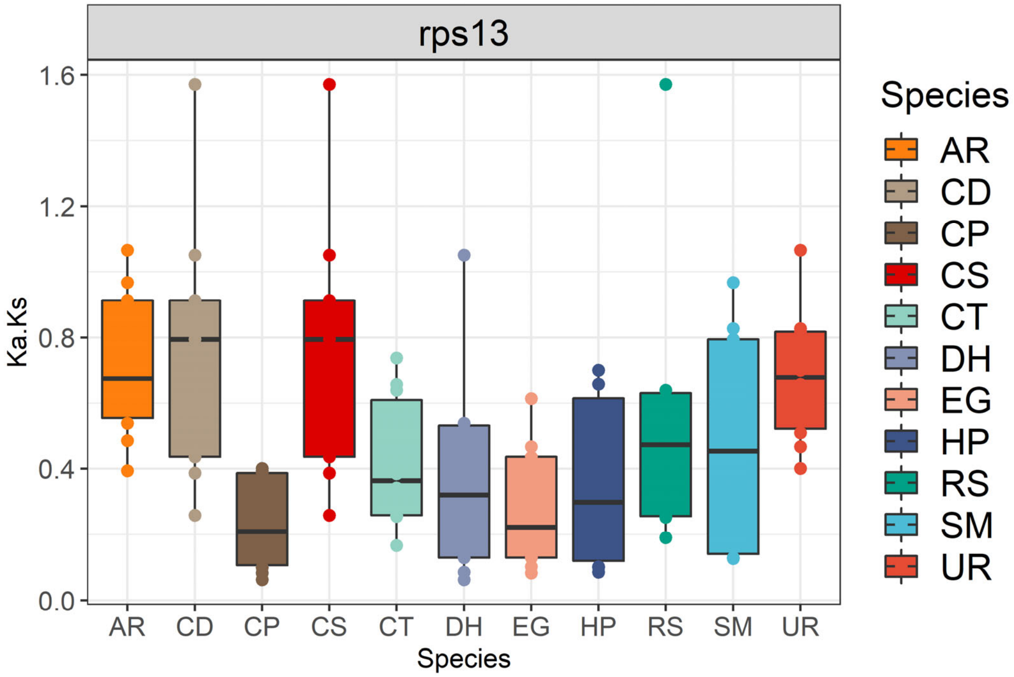

FIGURE S30 | Boxplots of pairwise Ka/Ks values in *rps*13 gene within 11 Lamiales species. AR – *Ajuga reptans*; CD – *Cistanche deserticola*; CP – *Castilleja paramensis*; CS – *Cistanche salsa*; CT – *Cistanche tubulosa*; DH – *Doroceras hygrometricum*; EG – *Erythranthe guttata*; HP – *Hesperelaea palmeri*; RS – *Rotheca serrate*; SM – *Salvai miltiorrhiza*; UR – *Utricularia reniformis*.

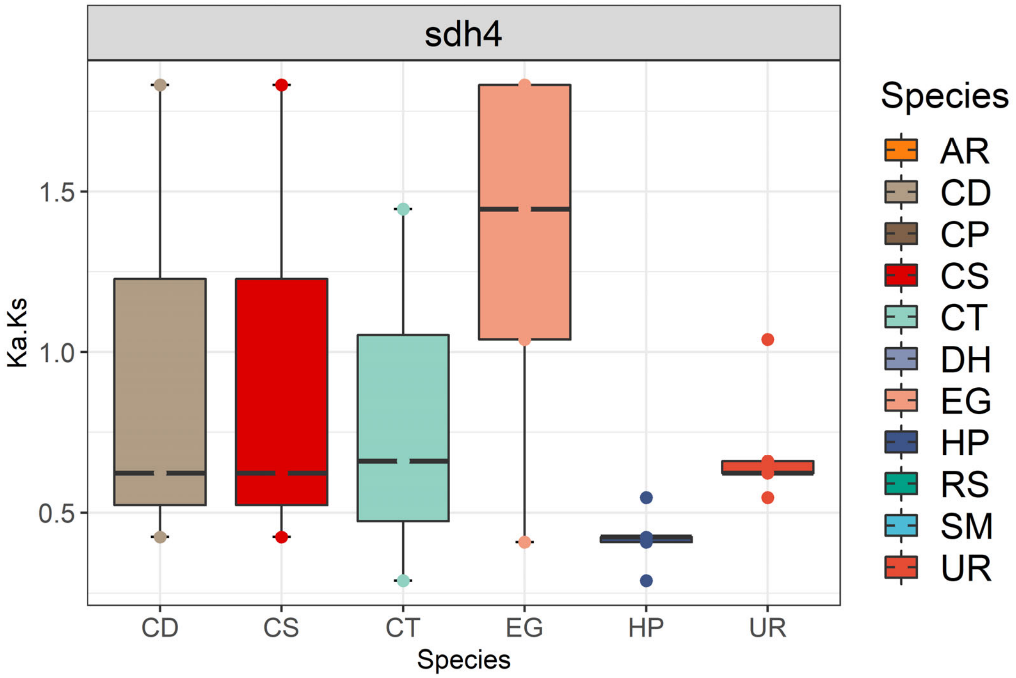

FIGURE S31 | Boxplots of pairwise Ka/Ks values in *sdh*4 gene within 11 Lamiales species. AR – *Ajuga reptans*; CD – *Cistanche deserticola*; CP – *Castilleja paramensis*; CS – *Cistanche salsa*; CT – *Cistanche tubulosa*; DH – *Doroceras hygrometricum*; EG – *Erythranthe guttata*; HP – *Hesperelaea palmeri*; RS – *Rotheca serrate*; SM – *Salvai miltiorrhiza*; UR – *Utricularia reniformis*.
